# TRPC4 Mediates Trigeminal Neuropathic Pain via Ca^2+^‐ERK/P38‐ATF2 Pathway in the Trigeminal Ganglion of Mice

**DOI:** 10.1111/cns.70368

**Published:** 2025-04-09

**Authors:** Xinlong Ke, Huajing Cai, Fangla Luo, Xing Zheng, Qian Hu, Youfa Zhou, Yongjie Wang, Xiangnan Zhang, Yeru Chen, Gang Chen

**Affiliations:** ^1^ Department of Anesthesiology, Sir Run Run Shaw Hospital, School of Medicine Zhejiang University Zhejiang Hangzhou China; ^2^ School of Pharmacy Hangzhou Normal University Zhejiang Hangzhou China; ^3^ Institute of Pharmacology & Toxicology, College of Pharmaceutical Sciences, Key Laboratory of Medical Neurobiology of the Ministry of Health of China Zhejiang University Zhejiang Hangzhou China; ^4^ Provincial Key Laboratory of Precise Diagnosis and Treatment of Abdominal Infection, Sir Run Run Shaw Hospital, School of Medicine Zhejiang University Zhejiang Hangzhou China

**Keywords:** ATF2, Ca^2+^, CION, ERK, P38, trigeminal neuropathic pain, TRPC4

## Abstract

**Background:**

Trigeminal neuropathic pain (TNP) is a debilitating condition characterized by chronic facial pain, yet its underlying mechanisms remain incompletely understood. Transient Receptor Potential Canonical 4 (TRPC4) has been reported to promote the development of abnormal pain or pain hypersensitivity in neuropathic pain. However, the specific contribution of TRPC4 to TNP pathogenesis remains unclear.

**Aim:**

This study aimed to investigate the role of TRPC4 in a mouse model of trigeminal neuropathic pain induced by chronic constriction of the unilateral infraorbital nerve (CION).

**Methods:**

Adult male/female mice were subjected to either CION surgery or sham surgery. Behavioral assays were conducted to assess facial pain‐like responses over a 28‐day period. TRPC4 distribution in the trigeminal ganglion (TG) was evaluated using Immunofluorescence. TRPC4 inhibitor ML204 and agonist Englerin A were employed to evaluate the impact of TRPC4 on facial pain‐like behaviors. A TRPC4‐overexpressing HEK293 cell model was conducted via plasmid transfection. To assess the function of TRPC4, we employed cellular calcium imaging technology to investigate the effects of modulating TRPC4 function by analyzing dynamic changes in intracellular calcium ion concentrations in primary trigeminal ganglion neurons and HEK293 cells. *Trpc4* shRNA was used to specifically knock down TRPC4 in the trigeminal ganglion. Western blot analysis was used to assess the activation of ERK, P38, and ATF2 signaling pathways.

**Results:**

Mice subjected to CION exhibited persistent facial pain‐like behaviors and a significant increase in TRPC4 expression in TG neurons. *Trpc4* shRNA or pharmacological inhibition with ML204 attenuated CION‐induced pain behaviors, while activation of TRPC4 with Englerin A induced pain‐like responses in naive mice. Calcium imaging revealed that both Englerin A and TRPC4 overexpression elevated intracellular Ca²^2+^ levels in TG neurons and HEK293 cells. This Ca²^2+^ influx triggered the activation of ERK and P38, leading to enhanced ATF2 activation. Downregulation of TRPC4 in the TG reduced ERK/P38 phosphorylation and ATF2 expression and activation.

**Conclusion:**

This study provides the first evidence that TRPC4 plays a critical role in CION‐induced trigeminal neuropathic pain by promoting the activation of the downstream transcription factor ATF2 via the Ca²^2+^‐ERK/P38 pathway.

## Graphical abstract

The specific mechanism of trigeminal neuropathic pain (TNP) is not fully understood. Through the chronic constriction of the unilateral infraobital nerve (CION) mouse model, we found that nerve compression injury leads to increased expression levels of TRPC4, a sensory neuron in the trigeminal ganglion. As a cation channel, TRPC4 functions to elevate intracellular calcium ion concentrations in sensory neurons, thereby activating the downstream ERK/P38‐ATF2 pathway and participating in trigeminal neuropathic pain. TRPC4 is expected to be a potential molecular target for the treatment of TNP.

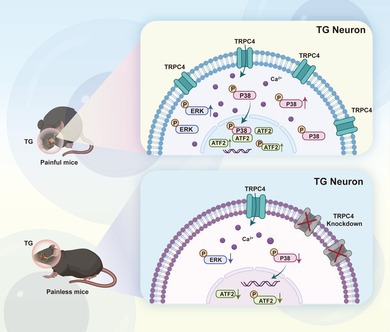

**Abbreviations**
ATF2: Activating Transcription Factor 2
CION: chronic constriction of the unilateral infraorbital nerve
Co‐IP: co‐immunoprecipitation
DRG: dorsal root ganglion
EA: Englerin A
ERK: extracellular signal‐regulated kinase
GAPDH: glyceraldehyde‐3‐phosphate dehydrogenase
HWT: head withdrawal threshold
ION: infraorbital nerve
JNK: c‐Jun N‐terminal kinase
MAPK: mitogen‐activated protein kinase
PBS: phosphate‐buffered saline
TG: trigeminal ganglion

## 1 Introduction

Trigeminal neuropathic pain is a medical disorder that occurs when the trigeminal nerve, particularly the V2 and/or V3 branches, is damaged, compressed, or affected by disease [1]. This pain can be elicited by mild sensory stimuli, such as touch, heat, and mechanical stimuli, on the affected side of the face. Patients may experience allodynia or other sensory alterations, which substantially influence their daily quality of life [2]. The treatment of TNP is frequently challenging, partly because of the ambiguous underlying pain mechanisms that arise from the original damage [1, 2, 3].

Transient receptor potential canonical (TRPC) channels represent a family of non‐selective cation channel proteins widely distributed in various cells of mammals, including vascular endothelial cells, neurons, and smooth muscle cells [4]. The mammalian TRPC channel family consists of seven members (TRPC1‐TRPC7), which can be divided into three subgroups: TRPC1/4/5, TRPC2, and TRPC3/6/7. In humans, apes, and monkeys, TRPC2 is considered a pseudogene and has lost its ability to encode functional proteins [5, 6]. In recent years, an increasing number of studies have reported that the TRPC channels play a crucial role in various pain models [7, 8, 9]. TRPC5 is closely related to the occurrence of inflammatory pain, such as pulpitis, arthritis, CFA‐induced inflammatory pain, and so forth [10, 11, 12], while TRPC6 is involved in chemotherapy‐induced peripheral neuropathy [9]. Specifically, TRPC4 is closely associated with neuropathic pain, morphine‐induced analgesic tolerance and hyperalgesia, migraine, visceral pain, and other pain conditions [13, 14, 15, 16]. Nevertheless, the current understanding of the involvement of TRPC in pain‐like behaviors generated by nerve damage is limited in models of TNP.

Mitogen‐activated protein kinases (MAPKs), including extracellular signal‐regulated kinase (ERK), c‐Jun N‐terminal kinase (JNK) and P38, are closely related to the development of chronic pain [17, 18]. Activating protein 2 transcription factors (ATF 2) are phosphorylated by mitogen‐activated protein kinases (MAPK) in their transactivation domains (TAD) at phosphorylation sites, which are a hallmark in response to growth factors, cytokines, or nerve injury [19]. Our research revealed that TRPC4 promotes TNP by elevating the levels of intracellular Ca^2+^ in sensory neurons, which consequently activates ERK and P38. This activation then triggers the transcription factor, ATF2. The present study provides the first evidence indicating that TRPC4 serves as a critical canonical channel to trigeminal neuropathic pain. Targeting the TRPC4‐ERK/P38‐ATF2 pathway in TG could offer a new therapeutic approach for managing trigeminal neuropathic pain.

## 2 Materials and Methods

### 2.1 Animals and Treatment

Male or female C57BL/6 mice, aged 10 weeks and weighing 23–25 g, were utilized. They were maintained in a 12‐h light/dark cycle with unrestricted access to water and food. All experiments were performed in adherence to the ethical guidelines set by the Animal Experiment Committee of Zhejiang University (Ethics Number ZJU20240451) and were in full compliance with the National Institutes of Health Guide for the Care and Use of Laboratory Animals. Measures were taken to reduce the number of animals used.

A similar mouse model of trigeminal neuropathic pain is established by chronic constriction of the unilateral infraorbital nerve (CION) via the intraoral approach as previously described [20, 21]. Briefly, mice were anesthetized with pentobarbital sodium at a dosage of 40 mg/kg (ip), and subsequently, the left masseter muscle tendon was identified on the upper wall of the oral cavity while the mice were in the supine position. An incision (1 mm) was made anterior to the tendon to expose the infraorbital nerve. The infraorbital nerve was ligated using 6‐0 black sterile silk suture to establish the TNP model. The mucosal incision was sealed using tissue adhesive. For sham surgery, the ION was not damaged, but an incision was made on the oral mucosa anterior to the left masseter muscle tendon. The entire surgery was performed under sterile conditions, and the mice were monitored, kept well‐hydrated, and maintained at a controlled temperature of approximately 37°C until they had fully recovered from anesthesia.

### 2.2 Drug Administration

The whiskers on the cheeks of the mice were shaved under anesthesia, and the mice were allowed to adapt for 3 days. After deep anesthesia with sevoflurane, one hand was used to hold the head of the mouse. Then, the needle tip was inserted through the infraorbital foramen, into the infraorbital canal, followed by the circular canal, and ultimately positioned within the trigeminal ganglion (TG) [22]. Then, 5 μL of the TRPC4 agonist Englerin A (0.25 μg; 2.5 μg and 25 μg, MCE) was injected. Then, 5 μL of PBS was injected as a vehicle control. As reported, the cheek injection model was used to measure pain‐related behaviors of different drugs. The whiskers on the cheeks of the mice were shaved under anesthesia, and the mice were allowed to adapt for 3 days. After that, 5 μL of the TRPC4 inhibitor ML204 (10 μg, MCE) was injected intracutaneously. Then, 5 μL of PBS was injected as a vehicle control. Seven days after the CION surgery, ML204 (2 mg/kg/day) (MCE) and vehicle solution were administered via intraperitoneal injection.

### 2.3 *Trpc4* shRNA Mice

As previously described, we administered viral injections into the trigeminal ganglia of mice [23]. Briefly, mice (6 weeks old) were anesthetized with pentobarbital sodium (40 mg/kg, ip), and the hair on the skull area of the mice was removed using a hair clipper and disinfected with a 1% povidone‐iodine solution. The mice were placed in a prone position on a stereotactic instrument, the bregma was exposed, and a microinjector was placed at the central position of the left trigeminal ganglion (TG) of the mice according to predetermined coordinates. The specific coordinates were: anteroposterior (AP) −4.5 ± 0.02 mm, mediolateral (ML) 1.6 ± 0.02 mm, dorsoventral (DV) 6.40 ± 0.04 mm. Using a glass micropipette, purified and concentrated adenovirus was slowly injected into the left trigeminal ganglion at a rate of 20 nL/min. After the injection, the glass micropipette was held in place for 20 min, then withdrawn from the TG, the scalp was sutured with sterile silk thread, and the mice were allowed to recover fully on a heating pad. Pain behavior tests were performed after allowing the mice to recover for 4 weeks. After the experiment, the injection site was verified histologically (see Figure S1). The CMV‐*Trpc4* shRNA adeno‐associated virus was constructed and packaged by Wuhan Shushu Brain Science and Technology Co. Ltd. (Wuhan, China). The shRNA sequence targeting *Trpc4* was 5'‐GCTCTCACCATCAGAGAAAGC‐3'; the negative control (scramble) shRNA was 5'‐GCCTAAGGTTAAGTCGCCCTC‐3'. The viral titer was > 5e+12 vg/mL.

### 2.4 Behavior Tests

#### 2.4.1 Mechanical Allodynia (Von‐Frey Test)

The “up‐down” method was employed to determine the mechanical pain threshold in the facial region of C57BL/6 mice [24]. Prior to testing, mice were allowed to acclimate to room temperature for at least 1 h. A set of seven Von Frey hairs, with forces logarithmically increasing (0.008, 0.02, 0.04, 0.07, 0.16, 0.4, and 0.6 g), was used to stimulate the infraorbital nerve region, specifically targeting the central area of the whisker pad located near the left upper lip (ipsilateral to the surgery). A brisk head withdrawal or paw touch to the left upper lip was considered a positive response. The “up‐down” method systematically increased and decreased stimulus intensity to determine the withdrawal threshold. Stimulation began with the 0.16‐g filament. The Von Frey filament was applied with sufficient force to cause slight bending and held for approximately 2–4 s. If no response occurred within 5 s, a heavier filament was used and recorded as “O”; if a positive response was elicited, a lighter filament was used and recorded as “X”. Each mouse's whisker pad was tested six times or until four consecutive positive or negative responses were obtained, with a 5‐min interval between each test. The threshold for facial mechanical pain sensitivity in mice was determined using the specific formula: 50% g threshold = [10^^^(*xf* + *kδ*)]/10,000 [25]. Here, *xf* represents the value of the final Von Frey filament used (in logarithmic units), *k* is the tabular value for the pattern of positive/negative responses, and *δ* is the average difference between stimuli (in logarithmic units, which is 0.296 in this case).

#### 2.4.2 Non‐Evoked Nociceptive Behavior

As mentioned earlier, compression injury to the infraorbital nerve can result in non‐evoked, persistent, or recurrent pain within the skin area innervated by the damaged nerve, manifesting as a behavioral response indicative of trigeminal neuropathic pain [25]. To evaluate alterations in spontaneous facial rubbing behavior, each mouse was placed individually within a transparent plexiglass cylinder (possessing a base radius of 5 cm and a height of 30 cm). Following a 30‐min adaptation period, the duration of rubbing behavior, specifically when the forelimbs contacted the ears or facial areas, was observed and recorded over a subsequent 30‐min period. According to the experimental methods of Gabriela Trevisan et al., compared to the sham surgery group, the facial grooming time of mice in the surgery group significantly increases [25].

#### 2.4.3 Cold Hypersensitivity

Cold hypersensitivity in C57BL/6 mice is evaluated by measuring acute nociceptive responses elicited by evaporative cooling induced by acetone [26]. In summary, each mouse is placed individually within a transparent plexiglass box (measuring 11 × 13 × 20 cm) positioned on an elevated metal mesh platform and allowed to habituate for at least 1 h prior to testing. A volume of 15 μL of acetone is carefully applied onto the skin surface of the left whisker pad (ipsilateral to the surgical site) of the mouse, and the duration of grooming behavior directed towards this area is measured for a period of 60 s. This application of acetone is repeated three times, with intervals of 10–15 min between each application, and the average nociceptive (grooming) time is subsequently calculated. Cold hypersensitivity is defined as an increase in nociceptive time observed after exposure to acetone, relative to baseline measurements (within the same animal) or compared to values obtained from sham‐operated animals (between different animals).

### 2.5 Cell Culture

#### 2.5.1 Isolation, Purification, and Culture of Mouse Trigeminal Ganglion Sensory Neurons

According to the previous method, isolate, purify, and culture mouse trigeminal ganglion sensory neurons [27]. Following anesthesia with isoflurane, the skull and brain of 6‐week‐old C57BL mice were promptly removed, and the trigeminal ganglia were quickly harvested [28]. Next, carefully remove the connective tissues around the ganglia, including blood vessels, fat, and nerve sheaths, in ice‐cold Hank's Balanced Salt Solution (HBSS). Use ophthalmic scissors to finely cut each ganglion into tissue fragments of approximately 0.5–1 mm^3^. Then, add 2 mL of 30 μg/mL papain solution (Worthington Biochemical, Cat. No. LS003126), incubate in a 37°C water bath for 20 min, centrifuge at 150 g for 1 min, and carefully remove the papain solution.

Afterwards, add 3 mL of a solution containing 4 mg/mL Dispase II (Sigma, Cat. No. D4693‐1G) and 4.5 mg/mL Collagenase II (Sigma, Cat. No. C2‐28‐100MG), and continue to incubate at 37°C for 20 min. Then, centrifuge at 400 *g* for 4 min to form tissue pellets and carefully remove the collagenase/dispase solution.

Add 1 mL of preheated B27 complete medium and 1.5 mg/mL DNase (Thermo Scientific, Cat. No. EN230AF) to the tissue, and gently pipette 2–3 times with a 200 μL pipette tip to disperse the cells. The procedure for preparing B27 complete medium is as follows: Mix neuronal basal medium (Gibco, Cat. No. 21103049) with B27 supplement (Gibco, Cat. No. 17504044), 1% penicillin/streptomycin (Solarbio, Cat. No. 240004004), 1% glutamine (Gibco, Cat. No. 35050061), and 50 ng/mL nerve growth factor (NGF, Cat. No. 450–34‐100UG) thoroughly. Next, prepare the Percoll gradient separation solution: Mix 1.2 mL of Percoll (YEASEN, Cat. No. 40501ES60) with 2.8 mL of preheated B27 complete medium to make a 30% Percoll solution, and mix 2.4 mL of Percoll with 1.6 mL of preheated HBSS to make a 60% Percoll solution. Carefully layer the 30% Percoll solution on top of the 60% Percoll solution.

Then, carefully layer 1 mL of cell suspension on top of the 30% Percoll solution, centrifuge at 1200 g for 10 min with slow deceleration. After centrifugation, collect the cells from the 30% layer and the 60%/30% interface into a 50 mL centrifuge tube. Add at least six times the volume of HBSS to the cell collection solution, centrifuge at 670 g for 4 min. At this point, cell pellets can be observed at the bottom of the tube. Finally, aspirate the medium and resuspend the cells in 600 μL of B27 complete medium. Inoculate the cell suspension into a confocal dish (NEST, Cat. No. 801001) precoated with poly‐D‐lysine and Laminin solution at a density of 1.0 × 10^5^ cells per milliliter. After allowing the cells to attach for 4 h, replace the medium with fresh B27 complete medium. Change half of the medium every 2 days. After 10 days of culture in vitro, these trigeminal ganglion (TG) neurons are used for calcium ion (Ca^2+^) imaging measurements, western blot and immunofluorescence staining.

#### 2.5.2 Cell Lines Culture

HEK293 cells were maintained in DMEM basic (1×) medium (Gibco, C11995500BT) containing 10% fetal bovine serum (EVERY GREEN, 23010702) and 1% penicillin/streptomycin (Solarbio, No. 240004004) in a humidity‐controlled incubator at 37°C with 5% CO_2_. HEK293 WT cells or TRPC4‐KO HEK293 cells, as well as primary trigeminal ganglion neurons, were treated with 1 μM Englerin A (MCE), an agonist of TRPC4, for 12 h. To inhibit p‐P38, primary trigeminal ganglion neurons were incubated with neuronal complete medium containing 1 μM SB202190 for 12 h. Total protein was extracted for western blot analysis. TRPC4‐KO HEK293 cells and WT HEK293 cells are from Guangzhou Yuanjing Biotechnology Co. Ltd.

#### 2.5.3 Plasmid Transfection

At 80% confluence, the cells were transfected with TRPC4 overexpression plasmid or control plasmid using jetPRIME Transfection reagent (Polyplus, No. 101000046). The maps of the TRPC4 overexpression plasmid and the control plasmid are shown in Figure S2. The transfected cells were maintained in the same growth medium for a duration of 40 h prior to Ca^2+^ measurement and Western blot analysis. Forty hours after transfecting HEK293 cells with the plasmid, the culture medium was replaced, and the cells were treated with the calcium chelator BAPTA‐AM at a concentration of 10 μM for 6 h. Following the 6‐h treatment, cell samples were collected for western blot analysis [29].

### 2.6 RNA‐Seq Data Processing

The trigeminal neuropathic pain dataset (GSE188922) was obtained from the Gene Expression Omnibus (GEO) database. Following data acquisition, fragments per kilobase of transcript per million mapped reads (FPKM) values were converted to transcripts per million (TPM) to facilitate downstream analyses. Differentially expressed genes (DEGs) were identified using the limma package, applying a significance threshold of *p* value < 0.05 and an absolute log2 fold change (|log2FC|) > 0.5. No correction for multiple comparisons was applied as the goal was to identify potential DEGs for further investigation. This analysis yielded 87 upregulated and 116 downregulated genes. To explore the potential involvement of these DEGs in relevant neuropathic pain, we performed keyword‐based searches using “neuropathic pain,” “migraine headache,” and “trigeminal neuropathic pain” in the Comparative Toxicogenomics Database (CTD) and GeneCards database. These searches identified 4555 and 3743 genes associated with the trigeminal neuropathic pain conditions, respectively. The VennDiagram package was subsequently used to identify the intersection of these gene sets, resulting in a shortlist of 16 candidate genes. To refine this selection and identify genes most strongly associated with the trigeminal neuropathic pain, least absolute shrinkage and selection operator (LASSO) regression was employed on the 16 candidate genes, yielding 4 genes with the highest predictive relevance. Data visualization, including the generation of volcano plots and heatmaps, was carried out using the ggplot2 and pheatmap packages, respectively.

### 2.7 RNA Isolation and Quantitative Real‐Time PCR Analysis

Total RNA from the trigeminal ganglion was extracted using the FastPure Cell/Tissue Total RNA Extraction Kit V2 (Vazyme Biotech Co. Ltd., Nanjing, China). The RNA quality and quantity were assessed using a NanoDrop spectrophotometer (Thermo Fisher Scientific, USA). Subsequently, cDNA was synthesized from RNA using the First Strand cDNA Synthesis Kit (Monad, China). Real‐time PCR was then performed using SYBR Green I (FOREGENE, China) on an A&B Applied Biosystems device. The expression levels of the target genes were standardized relative to *Gapdh* utilizing the 2ΔCt approach. The primer sequences of *Trpc1‐Trpc7* and *Gapdh* are provided in Table S1.

### 2.8 Western Blot

Using RIPA buffer (Beyotime, P0013B) containing protease inhibitor (YEASEN, 16403140) and phosphatase inhibitor cocktail (YEASEN, 10226191), the trigeminal ganglia from mice and HEK293 cells were homogenized. Protein concentration was quantified using the BCA protein concentration assay, and loading buffer (YEASEN, 201315ES20) was added. The samples were then heated at 95°C for 5 min to denature the proteins. From each sample, 25 micrograms of protein were separated by SDS‐PAGE (sodium dodecyl sulfate‐polyacrylamide gel electrophoresis) and transferred onto PVDF (polyvinylidene fluoride) membranes. After blocking with 5% non‐fat milk (Epizyme, PS112L) for 1 h, the membranes were incubated with the corresponding primary antibodies at 4°C for 16 h. A range of primary antibodies was utilized, including TRPC4 (1:1500; Alomone Labs, #ACC‐018 and Abclonal, A6996), p‐mTOR (1:1500; Abclonal, AP0115), mTOR (1:1500; Abclonal, A2445), p‐Akt (1:1500; Abclonal, AP1208), Akt (1:1500; Abclonal, A18675), p‐PI3K (1:1500; Cell Signaling Technology, 4292S), PI3K (1:1500; Abclonal, A4992), p‐ERK1/2 (1:1500; Abclonal, 83010S), ERK1/2 (1:1500; Abclonal, A4782), p‐P38 (1:1500; Abclonal, AP502), P38 (1:1500; Abclonal, A5049), p‐JNK (1:1500; Abclonal, AP1337), JNK (1:1500; Abclonal, A4867), p‐ATF2 (1:1500; Abclonal, AP1051), ATF2 (1:1500; Abclonal, A22718), and GAPDH (1:10000; Abcam, AB181602). The secondary antibody used was HRP goat anti‐Rabbit IgG (1:10000; Abclonal, AS070). Digital images were analyzed using densitometry with ImageJ software.

### 2.9 Immunofluorescence

Mice were euthanized under anesthesia, subsequently perfused with ice‐cold physiological saline, and then fixed with 4% paraformaldehyde overnight. Subsequently, the TG tissue was subjected to a gradient dehydration process using 10%, 20%, and 30% sucrose solutions, each for 24 h at 4°C. The TG tissue was then sectioned into 12 μm thick slices using a cryostat and prepared for immunofluorescence analysis. The tissue sections were first washed three times with PBS, each wash lasting 5 min, followed by a 1‐h blocking step using blocking solution. They were then incubated for over 16 h at 4°C with primary antibodies, including TRPC4 (1:200; Alomone Labs, No. ACC‐018), IB4 (1:100, Sigma, No. L2895), CGRP (1:100, Abcam, No. ab81887), NF200 (1:100, Sigma, No. N0142), and NeuN (Abcam, No. ab104224). Afterwards, the sections were incubated with Alexa Fluor 488 or 594 fluorescent secondary antibodies (Abcam, AB150105 and AB150076) for 1 h, followed by incubation with DAPI (Abcam, AB285390). We utilized ImageJ software to quantify the size distribution of neurons and assess the colocalization of TRPC4 with NF200, CGRP, or IB4. Specifically, in each experimental group, we selected 9 sections from each of three wild‐type (WT) animals, pooled these sections together for analysis, to accurately determine the number of TRPC4‐positive neurons and their colocalization ratios with NF200, CGRP, and IB4‐positive neurons.

After fixing primary trigeminal ganglion neuronal cells with 4% PFA (Biosharp, No. BL539A), the cells were evenly covered with blocking donkey serum and blocked at room temperature for 30 min. Subsequently, the cells were incubated overnight at 4°C in the dark with primary antibodies NeuN (1:200, Abcam, No. ab177487) and β3‐tubulin (1:200, Abcam, No. ab78078). Following this, the fixed cells were incubated with Alexa Fluor 488 or 594 fluorescent secondary antibodies (Abcam, No. AB150105 and AB150076) for 90 min, and then further incubated with DAPI (Abcam, No. AB285390) for 30 min. Co‐staining of ATF2 (1:300, CST, No. 35031S), p‐P38 (1:300, CST, No. 4511S), and β3‐tubulin (1:200, Abcam, No. ab78078) was performed using a four‐color fluorescence kit (AFIHC024; Hunan Aifang Biotechnology Co. Ltd.) based on tyramide signal amplification (TSA) technology according to the manufacturer's instructions. Finally, they were observed using a confocal microscope (OLYMPUS IX83‐FV3000‐OSR, Japan).

### 2.10 Calcium Imaging

Trigeminal ganglion (TG) neurons were incubated with Fura‐4 AM (4 μM, No. 2090588, Invitrogen), and HEK 293 cells were incubated with Cal‐590 (4 μM, MX4535, MKBio) in a cell culture incubator at 37°C for 20 min in the dark. The confocal dishes containing TG neurons or HEK 293 cells were rinsed with Artificial Cerebrospinal Fluid (ACSF) (No. R22153, Shyuanye, China) and subsequently mounted on a total internal reflection fluorescence microscope (Olympus IX83, Japan). The fluorescence intensity was measured at excitation wavelengths of 491 nm and 561 nm using a computer‐controlled fluorescence spectrophotometer, with detections made at 1‐s intervals and recorded for a duration of 5 min. The digital images were saved onto a hard drive for subsequent offline analysis. The fluorescence intensity at these two wavelengths was recorded separately, serving as indicators of the relative intracellular Ca^2+^ levels.

### 2.11 Co‐Immunoprecipitation (Co‐IP) Assay

The universal Co‐Immunoprecipitation Kit (Abbkine; Cat#: KTD104‐CN) was used for the experiment. Collect primary trigeminal ganglion neuronal cells, mix them with non‐denaturing lysis buffer, and then perform homogenization. The lysate was then incubated on ice at 4°C for 5 min. After centrifugation at 14,000 ×g at 4°C for 10 min, the supernatant was collected. Protein A/G magnetic beads were incubated with either anti‐ATF2 (1:50; CST; 35031S), anti‐phosphorylated P38 (1:50; CST; 4511S), or rabbit control IgG at room temperature for 40 min. Subsequently, the lysate supernatant was added to the Protein A/G magnetic bead‐antibody complex and incubated overnight at 4°C. The magnetic beads were then collected using a magnetic separation rack, and the supernatant was removed. The precipitate was washed three times with 1× Wash buffer. Lastly, 1× SDS‐PAGE Loading Buffer was added, the mixture was thoroughly mixed, and heated at 100°C for 5 min to denature the complexes. Western blot analysis was then performed using anti‐ATF2 (1:1500; Abclonal; A22718) and anti‐phosphorylated P38 antibody p‐P38 (1:1500; Abclonal, AP502).

### 2.12 Statistical Analysis

The experimental data are presented as Mean ± SD, and statistical analysis was conducted using Graphpad Prism 9.0 software. All data underwent normality testing using the Shapiro–Wilk test. For comparisons between two groups of data, if the data exhibit a normal distribution, Student's *t*‐tests are used; if the data do not exhibit a normal distribution, unpaired Mann–Whitney tests are used. For significance analysis involving more than two groups, we utilized one‐way ANOVA or two‐way ANOVA, followed by Tukey's multiple comparisons test. *p* < 0.05 indicates statistical significance.

## 3 Results

### 3.1 TRPC4 Is Associated with Trigeminal Neuropathic Pain induced by the Chronic Constriction of the Unilateral Infraorbital Nerve (CION)

**FIGURE 1:**
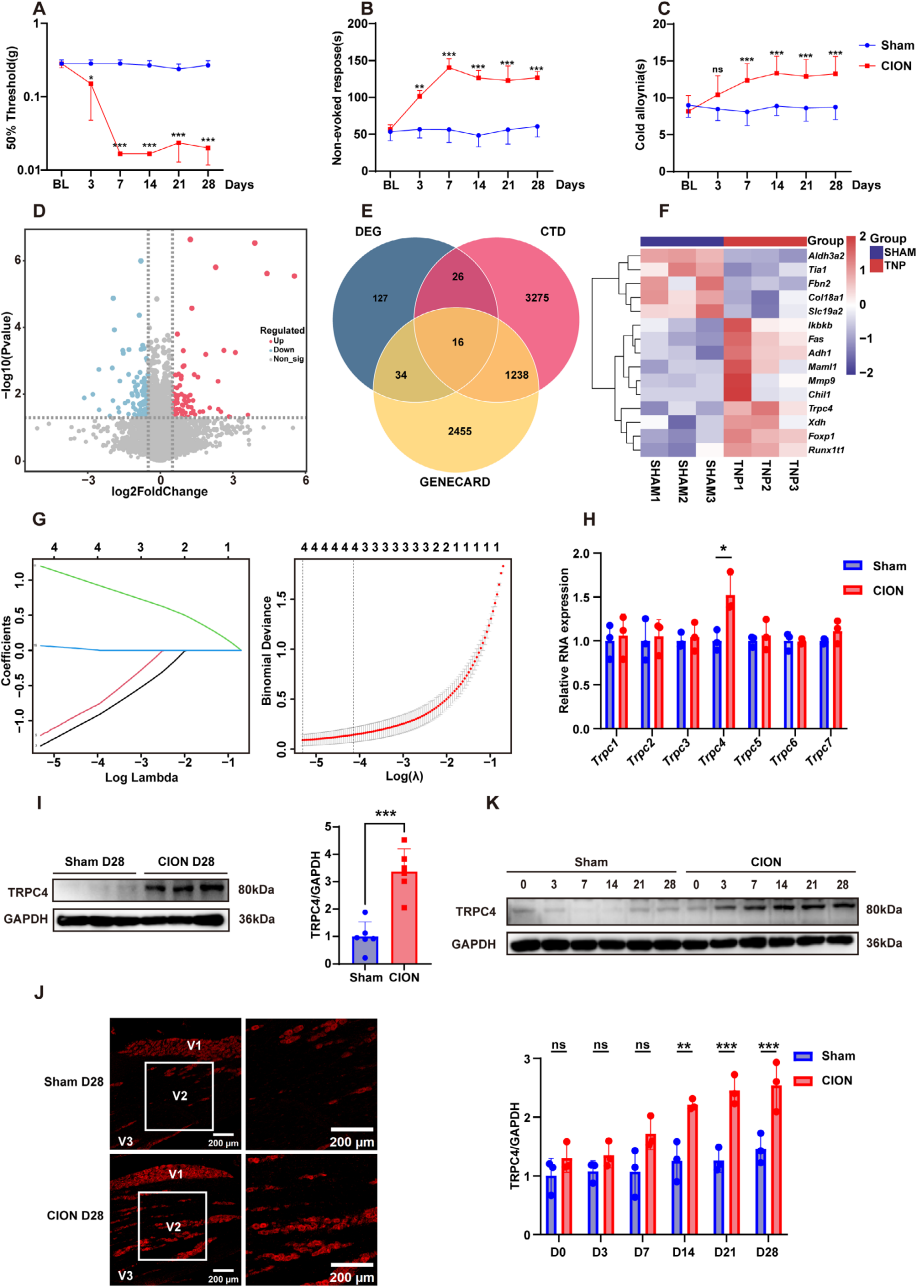
TRPC4 is associated with trigeminal neuropathic pain induced by the chronic constriction of the unilateral infraorbital nerve (CION). (A–C) In C57BL/6 male mice, constriction of the infraorbital nerve elicits mechanical allodynia (A), non‐evoked nociceptive behavior (B), and cold allodynia (C), persisting from day 3 until day 28 post‐surgery (*p < 0.05, **p < 0.01, and ***p < 0.001 versus sham, Two‐way repeated measured ANOVA, followed by Tukey's multiple comparisons test). (D) Volcano plot of DEG between SHAM and trigeminal neuropathic pain (*p* < 0.05, |Log2FC| > 0.5). (E) Venn diagram of the overlap between DEG, CTD, and GENCARD gene sets. (F) Heatmap results from transcriptome sequencing of the TG show increased *Trpc4* transcription levels after nerve injury. (G) Left: Coefficient plot showing the impact of variables on the expression of the intersection genes using LASSO regression. Right: Binomial deviance plot demonstrating the cross‐validation error rates across lambda values. (H) On the 28th day after CION surgery, real‐time fluorescent quantitative PCR was used to reveal the expression levels of *Trpc1*‐*Trpc7* mRNA in the trigeminal ganglia (TG) of both sham and CION mice, normalized to *Gapdh*. (*p < 0.05 vs. Sham; multiple unpaired Mann–Whitney tests; *n* = 6 mice/group). (I) The expression of TRPC4 in the ipsilateral trigeminal ganglion (TG) was assessed after CION or sham surgery, with normalization to GAPDH. (***p < 0.001 vs. sham, multiple unpaired Mann–Whitney tests; *n* = 6 mice/group). (J) On the 28th day after surgery, representative TRPC4 immunofluorescent images exhibit the distribution of TRPC4‐positive neurons within the trigeminal ganglion (TG) of both the sham surgery group and the CION group. (K) The expression level of TRPC4 in the trigeminal ganglion (TG) was measured after CION or sham surgery and normalized to GAPDH. *n* = 3 repeats. Data from three independent experiments. (multiple unpaired Mann‐Whitney tests. **p < 0.01 and ***p < 0.001 versus sham (Day 0), BL, baseline assessment before surgery).

The CION trigeminal neuropathic pain model was produced in adult male C57BL/6 mice by constriction of the infraorbital nerve (ION) according to the prescribed procedure. In the sham surgery group, the ION was only exposed without ligation. During the observation period from the third to the 28th day post‐surgery, we found that CION mice exhibited a decrease in the threshold for mechanical allodynia (Figure 1A), an increase in the duration of non‐evoked nociceptive responses (Figure 1B) and cold allodynia (Figure 1C). The above results of behavioral tests demonstrated the success of animal models in investigating trigeminal neuropathic pain. Furthermore, the analysis of the trigeminal neuropathic dataset revealed 87 upregulated genes and 116 downregulated genes, which were visualized using a volcano plot (Figure 1D). We conducted an intersection analysis between the differentially expressed genes (DEGs) and 4555 genes from the Comparative Toxicogenomics Database (CTD), together with 3743 genes associated with trigeminal neuropathic pain in GeneCards, to identify genes probable related to trigeminal neuropathic pain. This method ultimately found 16 candidate genes strongly linked to the disease (Figure 1E). To further refine the selection of potential genes, we utilized LASSO regression for further filtering (Figure 1F,G), ultimately identifying four candidate genes: *Adh1*, *Trpc4*, *Aldh3a2*, and *Slc19a2*. Considering the essential function of ion channels in the pathophysiology of neuropathic pain [30], and the lack of comprehensive research regarding the role of *Trpc4* in trigeminal neuropathic pain, *Trpc4* was selected for a further investigation. In order to verify the effect of TRPC4 on the CION surgery, we examined the alterations in TRPC family protein expression in the trigeminal ganglion (TG) after CION surgery. The real‐time fluorescent quantitative PCR results indicated a substantial increase in the mRNA level of *Trpc4* on the 28th day after CION surgery, while there were no significant changes observed in the mRNA levels of other members of the *Trpc* family (Figure 1H). Next, we assessed the expression of TRPC4 by western blotting assays. The findings revealed a substantial increase in the expression level of TRPC4 in the trigeminal ganglion of mice in the CION group, as compared to the sham surgery group (Figure 1I). There is a gender difference in the incidence of primary trigeminal neuralgia, with a higher prevalence in females, while traumatic trigeminal neuropathic pain exhibits no significant gender difference [1, 2]. Consequently, we have broadened our study sample to incorporate female mice. The results show that female mice also experience persistent facial pain following nerve injury, accompanied by an increase in TRPC4 expression in the trigeminal ganglion (Figure S3). The immunostaining results indicate notable disparities in the location of TRPC4‐positive neurons between the Sham and CION groups. Specifically, we observed a significant elevation in TRPC4 expression in the V2 region (Figure 1J). Given the detrimental impact of CION on the second branch of the trigeminal nerve and the role of the V2 region in orofacial sensory innervation, we hypothesize that the elevation of TRPC4 in the V2 region may significantly contribute to the neuropathic pain elicited by CION in this area of the mice. Furthermore, we detected the changes in TRPC4 expression over time after nerve injury. The findings demonstrated a large and sustained rise in TRPC4 expression in the trigeminal ganglion from the 14th to the 28th day following nerve damage (Figure 1K). The above results indicated that TRPC4 is relative to chronic constriction of the unilateral infraorbital nerve (CION) inducing trigeminal neuropathic pain.

### 3.2 TRPC4 Is Necessary and Sufficient for Trigeminal Neuropathic Pain

**FIGURE 2:**
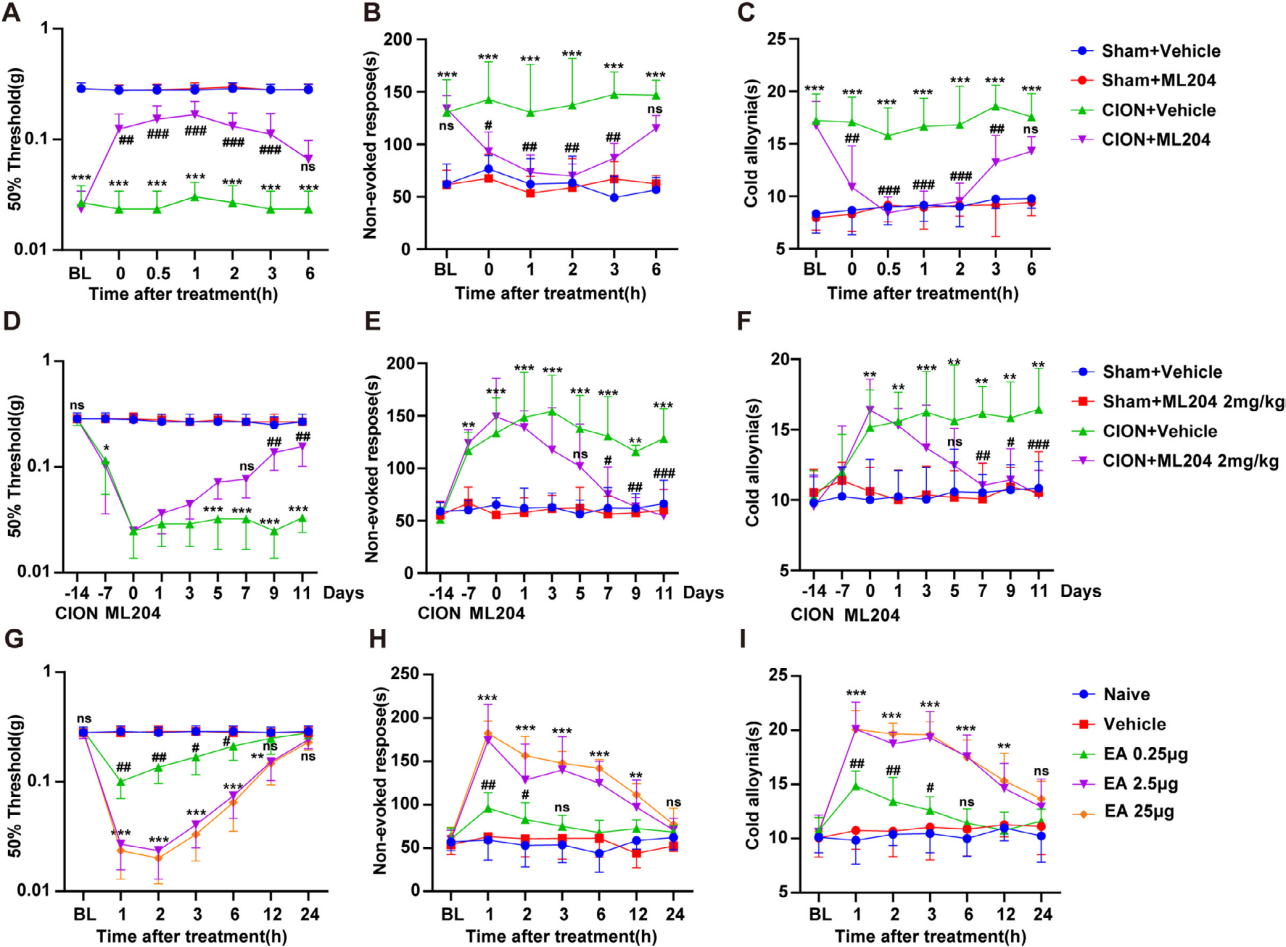
TRPC4 is necessary and sufficient for trigeminal neuropathic pain. (A–C) On the 14th day post‐surgery, a subcutaneous (*s.c*.) injection of ML204 (10 μg/5 μL) was administered into the left upper lip (ipsilateral to the chronic constriction of the infraorbital nerve (CION) surgery). The mechanical allodynia (A), spontaneous pain behavior (B) and cold allodynia (C) were measured before (BL) and after ML204 injection (Hour 0). (Two‐way repeated measured ANOVA, followed by Tukey's multiple comparisons test. *p < 0.05, **p < 0.01, and ***p < 0.001 versus Sham + Vehicle, #*p* < 0.05, ##*p* < 0.01, and ###*p* < 0.001 versus CION + Vehicle. *n* = 6 mice/group). (D–F) Effects of systemic administration of the selective TRPC4 receptor antagonist ML204 on mechanical allodynia (D), spontaneous pain behavior (E), and cold allodynia (F) following chronic constriction of the unilateral infraorbital nerve (CION). ML204 (2 mg/kg/day) was administered via intraperitoneal injection from Day 0 to Day 11, starting on Day 14 post‐CION. Measurements were taken during the period from post‐CION surgery to the administration of ML204. (Two‐way repeated measured ANOVA, followed by Tukey's multiple comparisons test. *p < 0.05, **p < 0.01, and ***p < 0.001 versus Sham + Vehicle. # *p* < 0.05, ## *p* < 0.01, and ### *p* < 0.001 versus CION + Vehicle, *n* = 6 mice/group). (G–I) The mechanical allodynia (G), spontaneous pain behavior (H) and cold allodynia (I) were measured at the first hour after injection of Englerin A (0.25 μg, 2.5 μg and 25 μg/5 μL) or vehicle solution PBS (5 μL) into the TG of WT mice, with a duration of 24 h. (Two‐way repeated measured ANOVA, followed by Tukey's multiple comparisons test. *p < 0.05, **p < 0.01, and ***p < 0.001 versus vehicle, *n* = 6 mice/group, BL, baseline assessment before surgery).

To detect the effect of TRPC4 on trigeminal neuropathic pain, we administered its specific inhibitor ML204 locally. On the 14th day after surgery, subcutaneous injection of ML204 was performed into the left upper lip on the same side. ML204 significantly alleviated trigeminal neuropathic pain induced by CION in the behavioral tests of mechanical allodynia (Figure 2A), non‐evoked pain response (Figure 2B), and cold allodynia symptoms (Figure 2C). Significantly, the administration of ML204 at the same dosage into the left upper lip of mice in the Sham control group did not have a noticeable impact on comparable pain‐related behaviors seen in their left upper lip (Figure 2A–C). Furthermore, administration of ML204 through intraperitoneal injection effectively alleviated mechanical hyperalgesia, non‐evoked pain behavior, and cold allodynia symptoms induced by infraorbital nerve compression (as shown in Figure 2D–F). We locally injected the agonist Englerin A into the trigeminal nerve ganglion (TG) and designed a dose‐gradient experiment consisting of three dose groups: 0.25 μg, 2.5 μg, and 25 μg. The experimental results showed that injecting Englerin A, a specific activator of TRPC4, into the TG could induce facial pain allodynia behaviors in mice similar to those observed in trigeminal neuropathic pain. Notably, as the drug dose increased, the intensity and duration of pain also exhibited a corresponding increasing trend. However, in animal behavioral observations, we found no significant difference in pain manifestations between the 2.5 μg and 25 μg dose groups (Figure 2G–I). Therefore, in subsequent experimental studies, we unanimously administered a dosage of 2.5 μg as the research dose. Collectively, these results clearly demonstrated that TRPC4 is essential in the generation and maintenance of pain sensations after nerve injury, and its specific pharmacological modulators hold promise as therapeutic agents.

### 3.3 Distribution of TRPC4 Protein in Trigeminal Ganglia of Naive Mice

**FIGURE 3:**
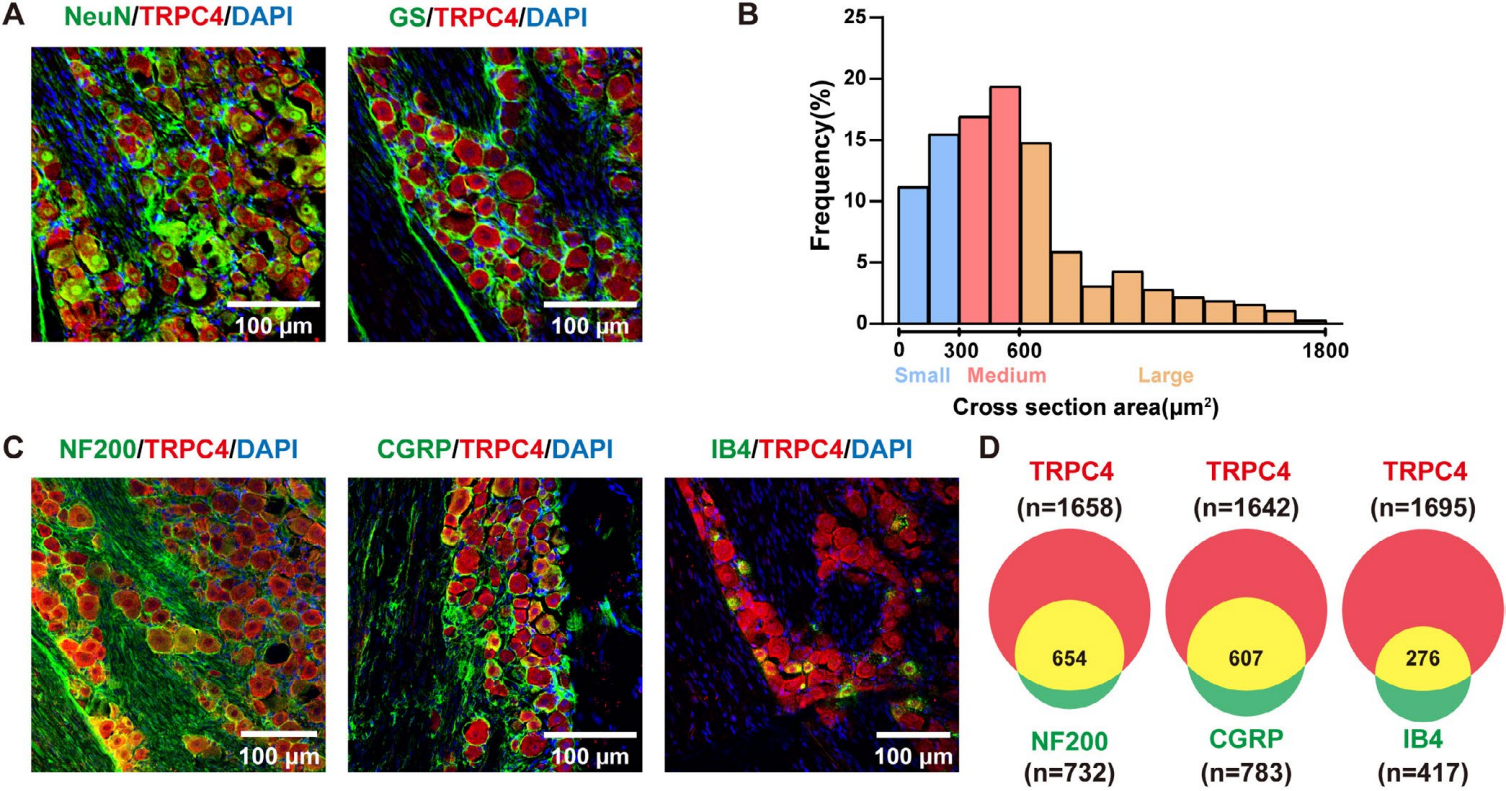
(A, B) Localization of TRPC4 immunostaining. (A) Double‐stained immunofluorescent images showing the co‐localization of TRPC4 with NeuN and GS. (B) Distribution of TRPC4‐positive somata: Large, 37%; medium, 36.3%; small, 26.7%. (C) Double‐stained immunofluorescent images showing the co‐localization of TRPC4 with NF200, CGRP, and IB4 in the TG. (Scale bars: 100 μm). (D) The Venn diagram shows the number of neurons double‐stained by TRPC4 and NF200, CGRP, or IB4. The experiment was conducted using 3 mice, and the data were derived from two independent experiments.

To investigate the potential involvement of TG TRPC4 in trigeminal neuropathic pain, we further analyzed its distribution pattern within the TG of mice. The results of dual immunostaining revealed that TRPC4 was exclusively colocalized with the neuronal marker NeuN, but not with glutamine synthetase (GS), a marker for satellite glial cells, suggesting that TRPC4 is expressed specifically in neurons (Figure 3A). Upon analyzing the cross‐sectional area of neuronal bodies, it was found that approximately 26.7% of TRPC4‐positive neurons were small (< 300 μm^2^), 36.3% were medium‐sized (ranging from 300 to 600 μm^2^), and 37% were large (> 600 μm^2^) (Figure 3B). In addition, we found that approximately 39.4% of TRPC4‐positive neurons were labeled by neurofilament‐200 (NF200, a marker for medium/large cells and myelinated Aβ‐fibers), 37.0% by calcitonin gene‐related peptide (CGRP, a marker for peptidergic neurons), and 12.2% by isolectin B4 (IB4, a marker for small non‐peptidergic neurons) (Figure 3C,D) (Figure S4).

### 3.4 TRPC4 Agonist Englerin A Increases the Ca^2+^ Concentration In Vitro

**FIGURE 4:**
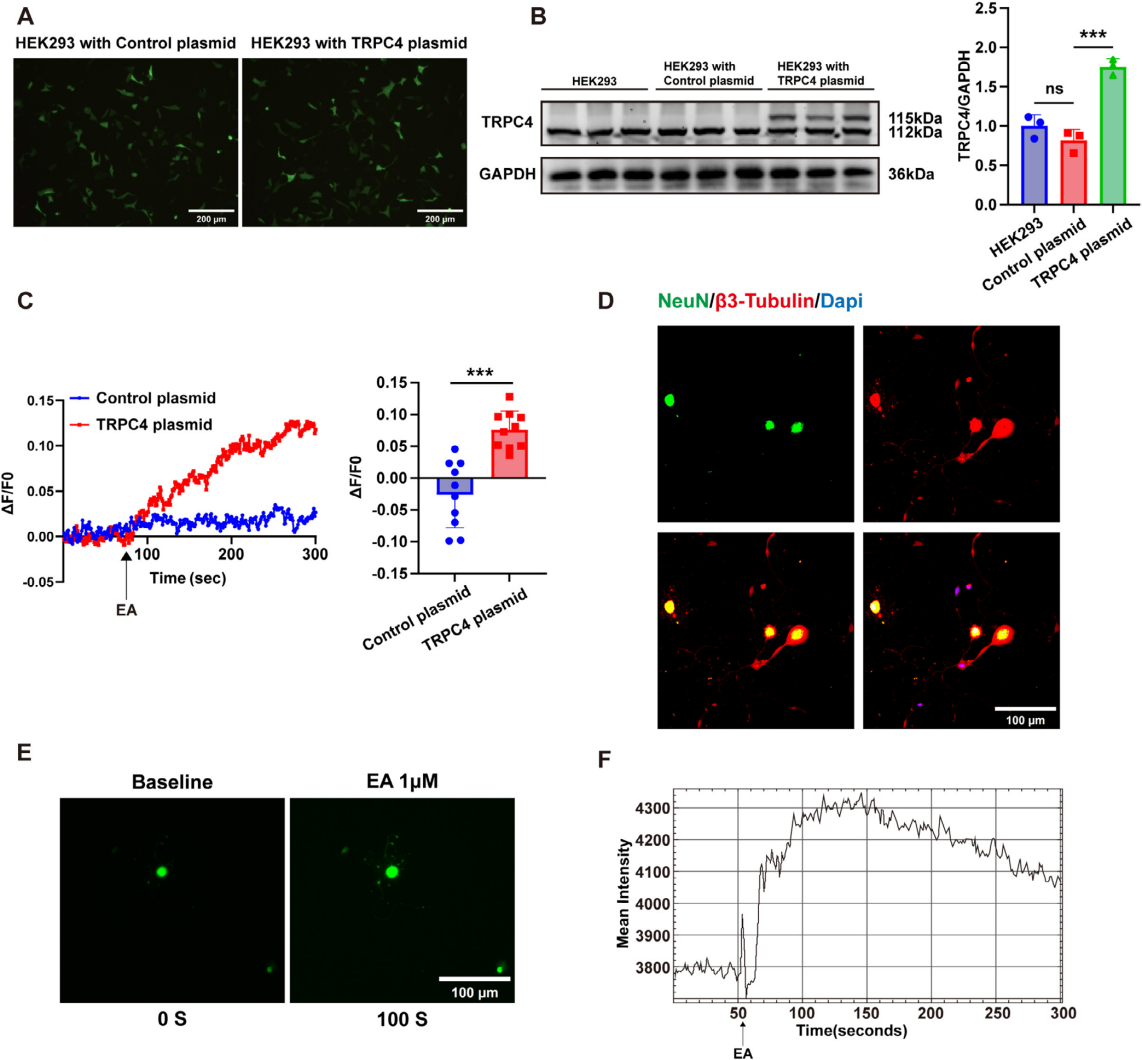
TRPC4 agonist Englerin A increases the Ca^2+^ concentration in vitro. (A) Example diagram of transfection of HEK293 cells with control plasmid and TRPC4 plasmids. (Scale bar: 200 μm). (B) The levels of TRPC4 protein were measured in HEK293 cells, in HEK293 cells transfected with control plasmids, and in HEK293 cells transfected with TRPC4 plasmids, with all measurements normalized to GAPDH. The sample size for each group is *n* = 3. (***p < 0.001, control plasmids vs. TRPC4 plasmids, one‐way ANOVA was used for statistical analysis, followed by post hoc Tukey test). (C) Effect of Englerin A on the ΔF/F0 ratio in HEK293 cells transfected with control plasmids or TRPC4 plasmids. Statistical data show that Englerin A increases the Δ*F*/*F*_0_ ratio in HEK293 cells transfected with TRPC4 plasmids, compared to HEK293 cells transfected with control plasmids. (***p < 0.001 control plasmids vs. TRPC4 plasmids; Student's *t*‐test; *n* = 10 cells/group). (D) Identification of primary trigeminal ganglion neurons using double‐stained immunofluorescent images of β3‐tubulin and NeuN. (Scale bar: 100 μm). (E) Representative Ca^2+^ images showing intracellular Ca^2+^ activity in trigeminal ganglion (TG) neurons at baseline and after application of Englerin A. (Scale bar: 100 μm). (F) Graph showing the change in mean fluorescence intensity of calcium signaling over time in neurons (shown above) after application of Englerin A. (Using ImageJ for plotting).

Intracellular calcium ions (Ca^2+^) function as a vital intracellular second messenger, engaging in multiple signaling pathways dependent on the availability of Ca^2+^ within the cell. Firstly, HEK293 cells were transfected with control plasmids and TRPC4 plasmids (Figure 4A). (The control plasmids and TRPC4 plasmids map is shown in Figure S1). Due to the overexpression plasmid carrying a 3 × flag tag, the molecular weight of exogenous TRPC4 is approximately 115 kDa, while the molecular weight of endogenous TRPC4 is 112 kDa. The western blot results confirmed the effect of TRPC4 plasmids in cells, exhibiting a significant rise in the expression level of total TRPC4 protein (Figure 4B). Further, we investigated the calcium response of HEK293 cells transfected with either control plasmids or TRPC4 plasmids upon exposure to Englerin A. The calcium imaging data revealed that treatment with 3 nmol/L of Englerin A had no observable effect on HEK293 cells transfected with control plasmids. However, it significantly increased the ΔF/F0 ratio in HEK293 cells transfected with TRPC4 plasmids (as shown in Figure 4C). Moreover, we successfully cultured primary trigeminal ganglion neurons in vitro and identified them using two neuron‐specific markers, NeuN and β3‐tubulin (Figure 4D). We further investigated whether Englerin A would affect the intracellular calcium ion concentration ([Ca^2+^]_*i*_) in primary trigeminal ganglion neurons. The findings demonstrated a significant and substantial rise in the Δ*F*/*F*_0_ ratio upon the addition of 1 μM of Englerin A to cultured primary trigeminal ganglion neurons. (*F*_0_ represents the average calcium signal value 30 s before drug administration, *F* represents the calcium signal value 30 s before the end of the experiment after drug administration, and Δ*F*/*F*_0_ = (*F*–*F*_0_)/*F*_0_). Our research results indicate that overexpression of TRPC4 by transfected with plasmids and pharmacological activation leads to an elevation in intracellular calcium ion concentration (Figure 4E,F).

### 3.5 The Activation of the AKT and MAPK Signaling Pathway by Englerin A Relies on the Presence of TRPC4

**FIGURE 5:**
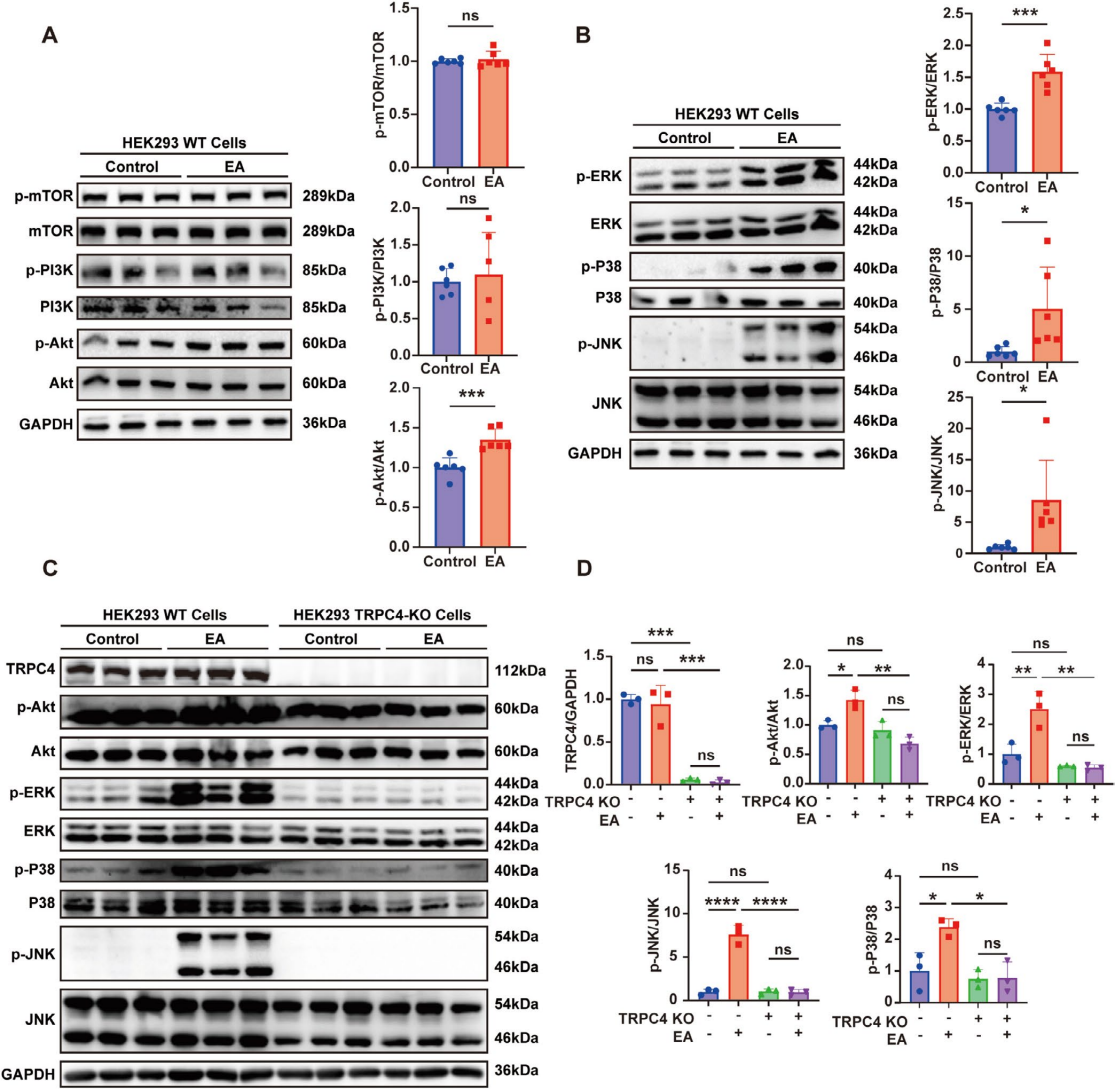
The activation of the AKT and MAPK signaling pathway by Englerin A is dependent on the presence of TRPC4. (A, B) The levels of p‐Akt, p‐ERK, p‐P38, p‐JNK, p‐PI3K, and p‐mTOR in HEK293 cells treated with EA were measured and normalized to GAPDH. (*p < 0.05, **p < 0.01, ***p < 0.001, control vs. EA; multiple unpaired Mann–Whitney tests, *n* = 6). (C, D) The levels of p‐Akt, p‐ERK, p‐P38, p‐JNK, p‐PI3K, and p‐mTOR in the TRPC‐KO cells and WT cells after treatment with Englerin A were measured and normalized to GAPDH. (Two‐way ANOVA followed by Tukey's multiple comparisons test. *p < 0.05, **p < 0.01, and ***p < 0.001; *n* = 3).

Calcium ions, as important second messengers, can activate multiple downstream signaling pathways, including the PI3K‐Akt signaling pathway and the MAPK signaling pathway. These two signaling pathways are particularly closely related to trigeminal neuropathic pain [31] (see Figure S5). In order to examine how these two pathways are involved in the effect of TRPC4 in neuropathic pain, we harvested the HEK293 cells after Englerin A treatment for 8 h. The results of western blot assays suggest that the expression levels of p‐Akt, p‐ERK, p‐P38, and p‐JNK were considerably higher in the Englerin A‐treated group compared with the control group (Figure 5A,B). However, there was no significant change in the expression levels of p‐PI3K and p‐mTOR (Figure 5A).

To further investigate whether Englerin A exerts its effects through TRPC4, TRPC4‐KO HEK293 cells were used for the subsequent experiment. The results of the western blot analysis revealed a notable reduction in the expression of TRPC4 in TRPC4‐KO HEK293 cells, indicating the successful generation of the TRPC4‐silenced cell lines (Figure 5C,D). We observed that treatment with Englerin A in wild‐type (WT) cells led to increased expression levels of p‐Akt, p‐ERK, p‐P38, and p‐JNK. Interestingly, Englerin A failed to activate the AKT and MAPK signaling pathways in the context of TRPC4 deficiency (Figure 5C,D). The data suggest that the activation of Akt, ERK, P38, and JNK by Englerin A is reliant on the presence of TRPC4.

### 3.6 The Sustained Activation of the AKT and MAPK Pathways Induced by TRPC4 Overexpression Is Dependent on Intracellular Calcium Ions

**FIGURE 6:**
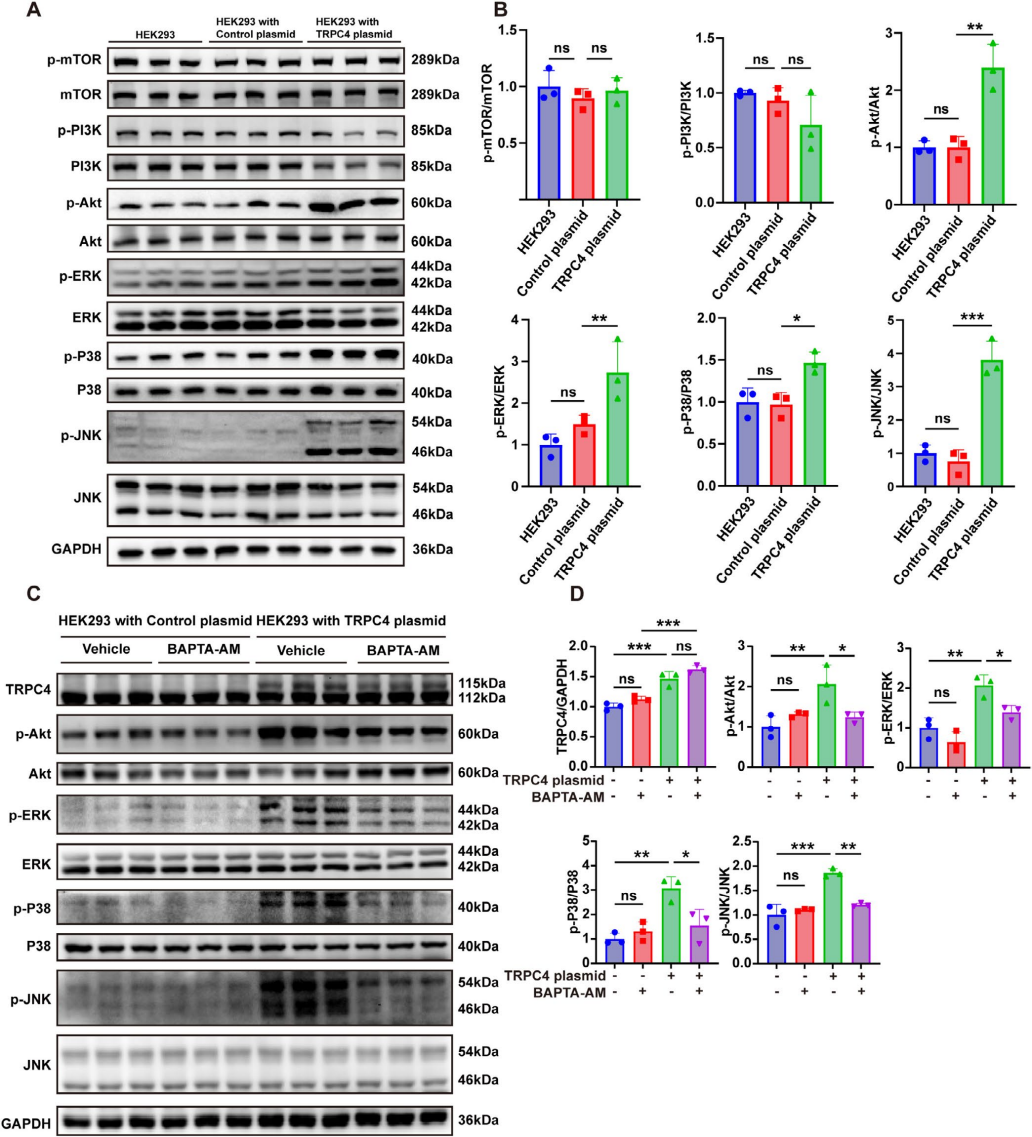
The sustained activation of the AKT and MAPK pathways induced by TRPC4 overexpression is dependent on intracellular calcium ions. (A, B) After transfection of HEK293 cells with TRPC4 plasmids, the expression levels of p‐mTOR, p‐PI3K, p‐Akt, p‐ERK, p‐P38, and p‐JNK were measured and normalized to GAPDH. (*p < 0.05, **p < 0.01, ***p < 0.001, control plasmids vs. TRPC4 plasmids, *n* = 3, one‐way ANOVA was used for statistical analysis, followed by post hoc Tukey test). (C, D) After transfecting HEK293 cells with TRPC4 plasmids and treating them with the calcium chelator BAPTA‐AM, the expression levels of p‐Akt, p‐ERK, p‐P38, and p‐JNK were measured and normalized to GAPDH. (Two‐way ANOVA followed by Tukey's multiple comparisons test. *p < 0.05, **p < 0.01, and ***p < 0.001; *n* = 3).

We further investigate the overexpression of TRPC4 induces alterations in the PI3K‐AKT pathway and the MAPK pathway. HEK293 cells transfected with TRPC4 plasmids exhibited a notable upregulation in the expression levels of p‐Akt, p‐ERK, p‐P38, and p‐JNK in comparison to cells transfected with control plasmids. However, there was no significant change observed in the expression levels of p‐PI3K and p‐mTOR (Figure 6A,B). The expression levels of these molecules did not alter when control plasmids were transfected into wild‐type (WT) HEK293 cells. The above results suggested an increase in TRPC4 expression leads to the activation of AKT and MAPK signaling pathways. To investigate whether the sustained activation of the AKT and MAPK pathways induced by TRPC4 overexpression depends on intracellular calcium ions, the HEK293 cells were transfected with TRPC4 plasmids to facilitate TRPC4 overexpression. Subsequently, the cells were administrated with the cell‐permeable calcium chelator BAPTA‐AM for 6 h. The results of western blot assays showed that BAPTA‐AM effectively reduced the activation of the AKT and MAPK signaling pathways induced by TRPC4 overexpression (Figure 6C,D). We conclude that the persistent activation of the ERK and P38 pathways induced by TRPC4 overexpression is dependent on intracellular calcium ions, and that TRPC4‐mediated calcium influx is essential for the activation of downstream MAPK pathways.

### 3.7 CION and Englerin A induce ERK and P38‐ATF2 Activation in the TG of Mice

**FIGURE 7:**
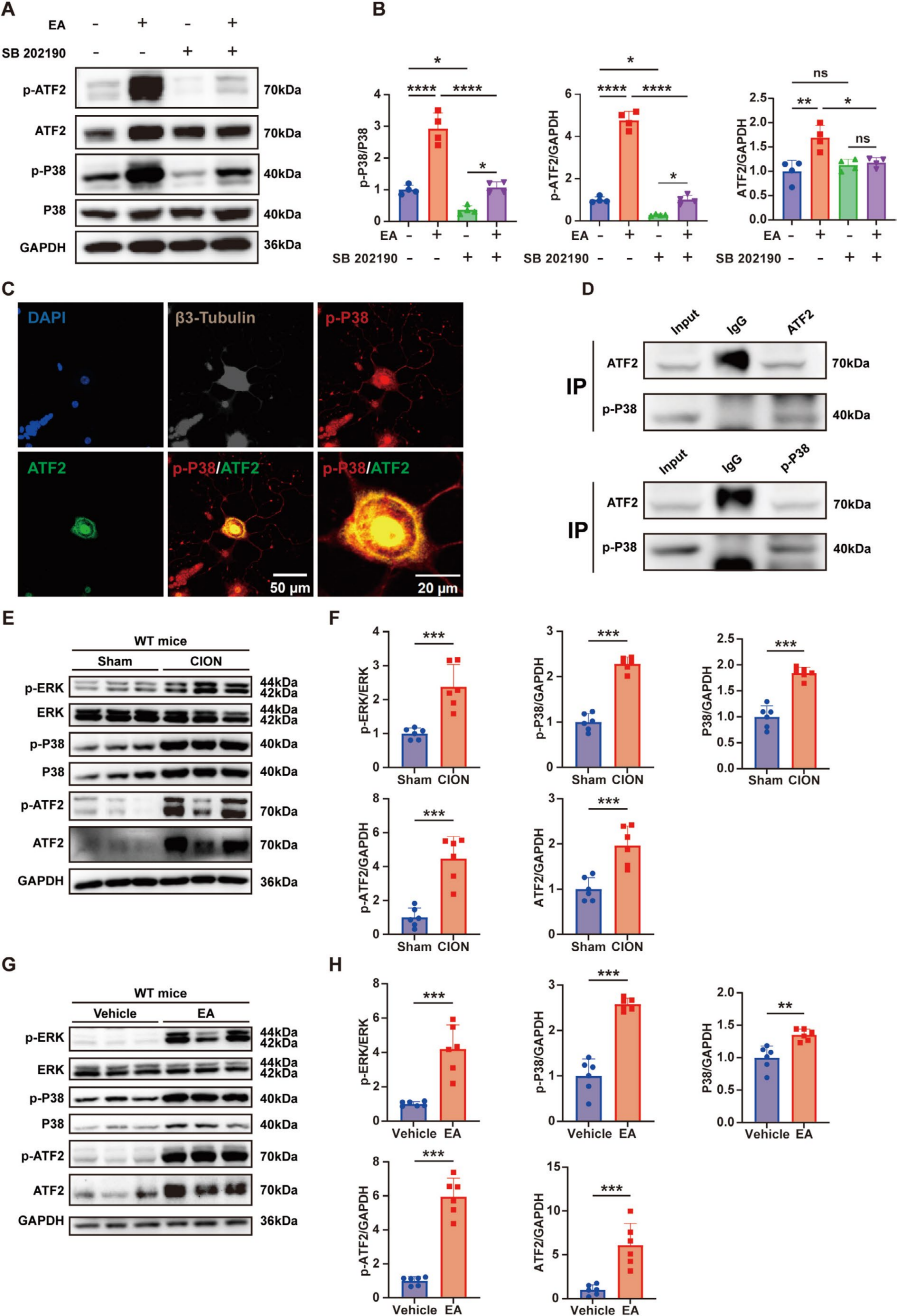
CION and Englerin A induce activation of ERK and P38‐ATF2 in the trigeminal ganglion of mice. (A, B) The expression levels of p‐P38, total P38, p‐ATF2, and total ATF2 in primary trigeminal ganglion neurons were measured after administration of Englerin A and SB 202190, and these levels were normalized to GAPDH. (Two‐way ANOVA followed by post hoc Tukey test. *p < 0.05, **p < 0.01, ***p < 0.001 and ****p < 0.0001, *n* = 4). (C) Triple immunofluorescence labeling images of ATF2, p‐p38, and β3‐Tubulin in primary trigeminal ganglion neurons. (Scale bar: 20 μm or 50 μm). (D) Western blot of ATF2 and p‐p38 immunoprecipitates from mouse primary trigeminal ganglion neurons. Normal rabbit IgG was used as a negative control. (E, F) Western blot analysis was performed to assess the protein levels of p‐ERK, p‐P38, P38, p‐ATF2, and ATF2 in the trigeminal ganglion (TG) of sham and CION mice groups, normalized to GAPDH. (***p < 0.001 vs. Sham, multiple unpaired Mann–Whitney tests; *n* = 6 mice/group). (G, H) Western blot analysis was performed to assess the protein levels of p‐ERK, p‐P38, P38, p‐ATF2, and ATF2 in the trigeminal ganglion (TG) of Vehicle‐treated and Englerin A‐treated mice groups, normalized to GAPDH. (**p < 0.01, ***p < 0.001 vs. Vehicle, multiple unpaired Mann–Whitney tests; *n* = 6 mice/group).

To further investigate the relationships between ATF2, TRPC4, and P38, we treated primary trigeminal ganglion neurons with 1 μM Englerin A for 12 h. Western Blot results indicated that Englerin A significantly increased the expression of p‐P38, ATF2, and p‐ATF2. SB 202190, a potent P38 MAPK kinase inhibitor, markedly reduced the downstream activation induced by Englerin A (Figure 7A,B). Subsequently, through immunofluorescence staining, we observed that p‐P38 colocalized with ATF2 in the nuclei of trigeminal ganglion neurons (Figure 7C), suggesting a potential interaction between p‐P38 and ATF2. Moreover, we confirmed a direct interaction between p‐P38 and ATF2 proteins by measuring co‐immunoprecipitation (Co‐IP) tests on mouse primary trigeminal ganglion neurons (Figure 7D). The above results indicate that p‐P38, as a downstream molecule of TRPC4, may directly act on the upstream of ATF2 and lead to its phosphorylation. Next, we further explored the relationship in CION mice. After WT mice received CION treatment, we observed a significant elevation in the expression levels of p‐ERK, p‐P38, P38, ATF2, and p‐ATF2 in the trigeminal ganglion (Figure 7E,F). However, there were no significant changes in the activation and expression levels of PI3K, Akt, mTOR, and JNK (Figure S6). These results indicate that MAPKs, not the Akt pathway, have a role in the pathological process of trigeminal neuropathic pain. To determine whether TRPC4 is involved in the PI3K‐AKT pathway and MAPK pathway in TG, we administered Englerin A into TG and assessed the expression of pivotal proteins in these two pathways. Interestingly, consistent with CION, intra‐TG injection of 2.5 μg/day Englerin A resulted in a rise in the expression of p‐ERK, p‐P38, P38, ATF2, and p‐ATF2 in the TG of WT mice (Figure 7G,H). Similarly, compared with the vehicle group, there were no notable differences in the activation and expression levels of PI3K, Akt, mTOR, and JNK in the TG following the administration of Englerin A (Figure S6). This suggests that stimulation of TRPC4 plays an important role in triggering the MAPK signaling pathway in TG, resulting in the development of trigeminal neuropathic pain.

### 3.8 Downregulation of TRPC4 in the Trigeminal Ganglion Alleviates Neuropathic Pain Triggered by CION Through ERK and P38‐ATF2 Pathway

**FIGURE 8:**
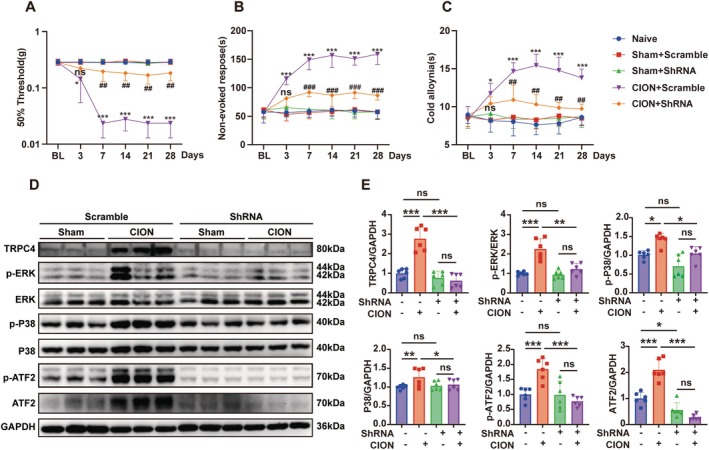
Downregulation of TRPC4 in the trigeminal ganglion alleviates neuropathic pain triggered by CION through the ERK and P38–ATF2 pathway. (A–C) On the 28th day after virus injection into the trigeminal ganglion (TG), the CION group mice underwent chronic constriction of the unilateral infraorbital nerve (CION) surgery, while the Sham group mice received sham surgery. The naive group mice did not receive any treatment. Mechanical hyperalgesia (A), spontaneous pain behavior (B), and cold allodynia (C) were measured in these mice before (BL) and after CION surgery (from Day 3 to Day 28). (D, E) Western blots and analysis of protein expression of TRPC4, p‐ERK, ERK, p‐P38, P38, p‐ATF2, and ATF2 in the Sham + Scramble groups, CION + Scramble groups, Sham + shRNA groups, and CION + shRNA groups, normalized to GAPDH. (Two‐way ANOVA, followed by Tukey's multiple comparisons test. *p < 0.05, **p < 0.01, and ***p < 0.001; *n* = 6 mice/group).

Given the existing limitation of TRPC4 inhibitors and activators, which lack specificity for TRPC4 and simultaneously affect TRPC5, we employed shRNA technology to specifically downregulate the expression of TRPC4 in the trigeminal ganglion of mice. Subsequently, we observed changes in facial pain behavior in *Trpc4* shRNA mice after injecting EA into the TG and CION, further validating the specific roles of TRPC4 and the MAPK pathway in the trigeminal ganglion. We used a stereotactic apparatus to inject *Trpc4* shRNA into the trigeminal ganglion of 6‐week‐old mice and allowed them to recover for 4 weeks. After establishing the CION model, we found that compared with the mice injected with Scramble virus, *Trpc4*‐silencing virus in the trigeminal ganglion alleviated mechanical allodynia (Figure 8A), non‐evoked nociceptive responses (Figure 8B), and cold allodynia (Figure 8C). It is noteworthy that there were no differences in pain behavior among the sham group injected with Scramble virus, the sham group injected with *Trpc4* shRNA, and the untreated naive group. After the behavioral tests, we harvested the trigeminal ganglion tissues 28 days after CION treatment for protein quantification and immunofluorescence analysis. The immunofluorescence staining results showed that the fluorescence intensity and quantity of TRPC4 protein were significantly reduced in the mice injected with *Trpc4* shRNA (Figure S1A). Here, we paid particular attention to the targeting specificity of the *Trpc4* shRNA virus. RT‐qPCR tests revealed that following CION modeling, *Trpc4* shRNA selectively reduced the mRNA level of *Trpc4* in the trigeminal ganglion without influencing the mRNA levels of other Trpc members (Figure S1B). The western blotting experiments provided additional evidence that *Trpc4* shRNA effectively suppressed the elevated expression of TRPC4, p‐ERK, p‐P38, P38, p‐ATF2, and ATF2 induced by CION (Figure 8D,E).

### 3.9 Downregulation of TRPC4 in the Trigeminal Ganglion Mitigates Facial Pain‐Like Behaviors Triggered by Englerin A via the ERK and P38‐ATF2 Pathways

**FIGURE 9:**
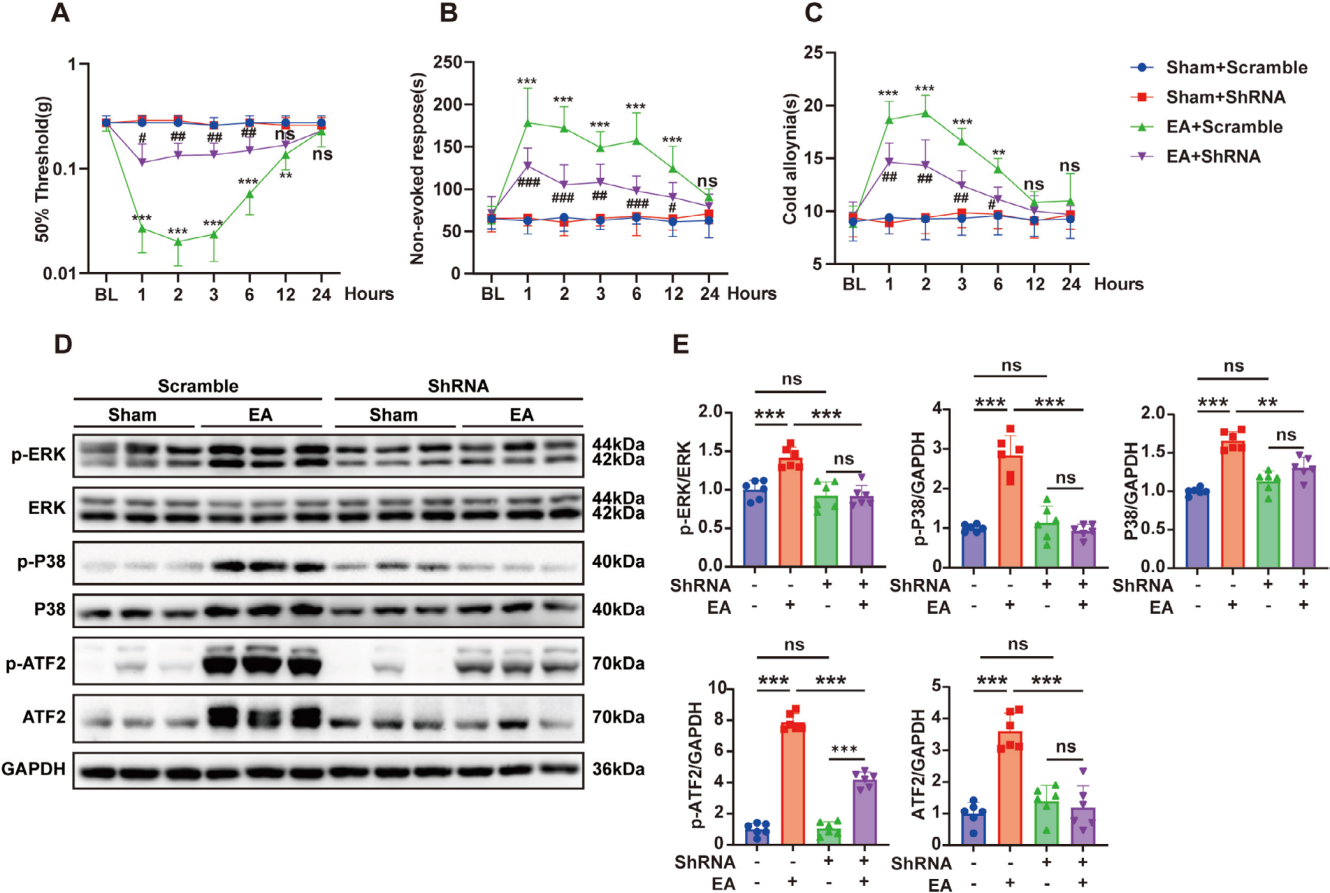
Downregulation of TRPC4 in the trigeminal ganglion mitigates facial pain‐like behaviors triggered by Englerin A via the ERK and P38‐ATF2 pathways. (A–C) On the 28th day after virus injection into the trigeminal ganglion (TG), the Englerin A group mice were injected with EA into the TG, while the Sham group mice were injected with control solution into the TG. Mechanical hyperalgesia (A), spontaneous pain behavior (B) and cold allodynia (C) were measured in these mice before (BL) and after intra‐TG injection of Englerin A (from Hour 1 to Hours 24). (D, E) Western blots and analysis of protein expression of p‐ERK, ERK, p‐P38, P38, p‐ATF2, and ATF2 in the Sham + Scramble groups, Englerin A + Scramble groups, Sham + shRNA groups, and Englerin A + shRNA groups, normalized to GAPDH. (Two‐way ANOVA, followed by Tukey's multiple comparisons test. *p < 0.05, **p < 0.01, and ***p < 0.001; *n* = 6 mice/group).

Furthermore, we also found that downregulation of TRPC4 in the trigeminal ganglion could alleviate mechanical allodynia (Figure 9A), non‐evoked nociceptive responses (Figure 9B), and cold hyperalgesia (Figure 9C) induced by Englerin A. There were no differences in pain behavior between the sham group injected with control virus and the sham group injected with TRPC4 shRNA. Further evidence that TRPC4 shRNA might decrease the enhanced levels of TRPC4, p‐ERK, p‐P38, P38, p‐ATF2, and ATF2 brought about by Englerin A was provided by the outcomes of western blotting assays (Figure 8D,E). This indicates that downregulating TRPC4 in the trigeminal ganglion can alleviate neuropathic pain triggered by CION and Englerin A through the ERK and P38‐ATF2 pathway.

## 4 Discussion

Trigeminal neuropathic pain (TNP) is the pain caused by damage or injury of the trigeminal nerve, particularly the V2 and/or V3 branches. Recent investigations in general practitioner (GP) databases have shown a significant increase in the incidence of trigeminal neuropathic pain (TNP), with rates ranging from 28 to 12 per 100,000 individuals [32]. Furthermore, emerging data suggest that TNP may be more prevalent among younger age groups [33]. The underlying mechanisms of TNP have been challenging to ascertain. The present work elucidates the significant contribution of TRPC4 in the development of Trigeminal neuropathic pain. Targeting the TRPC4‐ERK/P38‐ATF2 pathway in the trigeminal ganglion (TG) could provide a new and effective therapeutic approach for the management of trigeminal neuropathic pain.

Transient receptor potential channels (TRPCs) are a class of non‐selective cation channels primarily located on the plasma membrane of several human and animal cell types. They have the ability to react to various stimuli, including temperature, pH changes, osmotic pressure changes, and inflammatory mediators. They participate in multiple physiological and pathological processes within the body [5]. Prior studies have mostly emphasized the involvement of TRPA1 and TRPV1 in chronic pain, but limited study has been conducted on the role of TRPCs in pain. In this study, we found that chronic constriction of the unilateral infraorbital nerve (CION) causes trigeminal neuropathic pain‐like behavior. Additionally, there is an increase in TRPC4 expression in the trigeminal ganglion (TG) under this condition, and this rise is directly associated with neuropathic pain‐like behavior (Figure 1). However, we did not observe changes in the expression of other TRPCs after CION (Figure 1H). There is a lack of previous reports regarding the specific location of TRPC4 in the TG. This study demonstrated that TRPC4 is co‐expressed with neuronal NeuN, while it is not detected in GS‐positive satellite cells (Figure 3A). TRPC4 is co‐localized with CGRP, NF200, or IB4, which are exclusively present in TG neurons (Figure 3C). Given that NF200+, CGRP+, and IB4+ neurons project C‐ and Aβ‐afferent fibers in the peripheral nervous system and are commonly acknowledged as conveying painful signals to pain centers, it can be inferred that TRPC4 may play a role in the processing of pain signals.

Pharmacological inhibitors ML204 and agonist Englerin A for TRPC4 have been developed previously [4]. The selective local and systemic administration of the TRPC4 antagonist ML204 improved trigeminal neuropathic pain‐like behavior after CION treatment. Furthermore, repeated systemic treatment with ML204 during nerve injury can cause a delay in the emergence of pain‐like behavior, but it cannotentirely prevent it; this suggests that for the prospective therapeutic application of TRPC4 antagonists, long‐term treatment techniques need to be taken into account (Figure 2D–F). Local administration of ML204 was found to exclusively reduce pain‐like behavior when injected on the same side as the injury, indicating that the effects of TRPC4 targeting are specific to the injured nerve (Figure 2A–C). According to previous methods, intra‐TG injection of the TRPC4 agonist Englerin A can lead to facial pain‐like behavior, manifested as mechanical hyperalgesia (Figure 2G), non‐evoked pain behavior (Figure 2H), and cold allodynia symptoms (Figure 2I). The crucial role of TRPC4 in maintaining hyperalgesia induced by CION is further confirmed by pharmacological research results. Given the lack of specificity of existing TRPC4 inhibitors and activators, which simultaneously affect TRPC5 [34], we employed shRNA technology to specifically downregulate the expression of TRPC4 in the trigeminal ganglion of mice. Further research has found that downregulation of TRPC4 in the TG can reduce neuropathic pain induced by CION and Englerin A. This suggests that TRPC4 contributes to neuropathic pain.

The mitogen‐activated protein kinase (MAPK) is an evolutionarily conserved protein family that is crucial for intracellular signal transduction and plays a key role in regulating neuropathic pain [17]. The MAPK family consists of three main members: extracellular signal‐regulated kinase (ERK), P38, and c‐Jun N‐terminal kinase (JNK), which represent three independent signal transduction pathways. The ERK signaling pathway is crucial for the development and regulation of pain, and its activation can promote central sensitization [35, 36]. In various animal models of chronic pain, such as chronic nerve compression [37], spinal injury [38], spinal nerve injury [39], and morphine‐induced pain hypersensitivity [14], ERK is activated in nociceptive primary sensory neurons and spinal neurons. Intrathecal injection of the P38 inhibitor SB203580 can alleviate inflammatory pain [40, 41], bone cancer pain [42], incision pain [43], and morphine‐induced pain hypersensitivity [44]. Similarly, injection of the P38 inhibitor SB203580 into the trigeminal ganglion (TG) can alleviate tongue pain and CION‐induced trigeminal neuropathic pain (TNP) [45, 46]. Activation of P38 MAPK can lead to upregulation of various inflammatory mediators (cyclooxygenase 2, iNOS, TNF‐α, IL‐1β). Additionally, it can affect the expression and function of specific ion channels, including NaV1.7, NaV1.8, TRPV1, and TRPA1, which in turn regulate the sensitivity of sensory neurons and their responses to noxious stimuli [18]. In our research, we found that both CION and injection of EA into the trigeminal ganglion led to increased expression of p‐ERK, p‐P38, and P38. However, JNK did not show any activation. This finding aligns with earlier studies indicating that CION does not activate JNK in the MAPK signaling pathway [35]. In addition, we have noted that apart from TRPC4, molecules such as voltage‐gated sodium channels (VSGCs), Toll‐like receptor 8(TLR8), and chemokine CXCL13 also contribute to the pathogenic mechanism of trigeminal neuropathic pain through the MAPK pathway [22, 35, 47]. The increase in intracellular Ca^2+^ is crucial for activating MAPK (ERK and P38) [17, 48]. Calcium imaging results showed that the TRPC4 agonist Englerin A can lead to an increase in intracellular calcium ion concentration in trigeminal ganglion (TG) neurons and HEK293 cells transfected with TRPC4 plasmid. The increased intracellular calcium ions can arise from the entry of extracellular Ca^2+^ influx or release from organelles such as the endoplasmic reticulum and lysosomes [49, 50]. In vitro experiments, due to the presence of Ca^2+^ in the Artificial Cerebrospinal Fluid (ACSF) used, the increase in intracellular calcium ion concentration induced by Englerin A originates primarily from the extracellular environment. Subsequently, it leads to a prolonged and sustained high level of intracellular calcium ion concentration, which may also be sourced from intracellular organelles such as the endoplasmic reticulum or lysosomes [48, 51]. Additional investigation is required to comprehend the mechanism by which TRPC4 facilitates the release of intracellular calcium.

ATF2, located in the dorsal root ganglion (DRG) and spinal dorsal horn, has been shown to be involved in maintaining neuropathic pain caused by nerve injury [19]. The transcription factor ATF2 is likely to operate as a downstream mediator of P38 in the regulation of genes [17]. Nevertheless, the precise role of ATF2 in trigeminal ganglion (TG) neurons remains to be elucidated. Our research has found that phosphorylated ATF2 (p‐ATF2) is mostly present in the nuclei of primary trigeminal ganglion neurons. Furthermore, the activation of TRPC4 results in higher levels of p‐ATF2 expression, indicating that ATF2 is a downstream signaling molecule that is triggered by TRPC4. The results of co‐immunoprecipitation (Co‐IP) assays demonstrated that there was an interaction between p‐P38 and ATF2 in mouse trigeminal ganglion neurons (Figure 7C,D). Under chronic constriction of the unilateral infraorbital nerve (CION) and Englerin A treatment, the activated P38 translocates to the nucleus and binds with the transcription factor ATF‐2, leading to the phosphorylation of ATF2. Previous studies have shown that ATF2 can bind to c‐Jun and together regulate the expression of multiple targets [52, 53]. Specifically, c‐Jun has been found to regulate the expression of vasoactive intestinal peptide (VIP) and neuropeptide Y (NPY) in cultured DRG neurons of neuropathic rats [54]. According to these details, it is speculated that the ATF2/c‐Jun heterodimer might enhance the production of VIP and NPY, leading to an increase in pain perception in the DRG and spinal cord following damage [54]. Nevertheless, ATF2 may possess additional targets that play a role in pain perception in neuropathic mice, which is a crucial avenue for our forthcoming investigation.

It is particularly noteworthy that a previous study reported a decrease in *Trpc4* mRNA levels in the dorsal root ganglion (DRG) following spared nerve injury of the sciatic nerve [55]. Our findings, however, differ from the changes in TRPC4 expression observed in that study, likely due to the combined effects of multiple factors, including the significant differences in the neural structures investigated (TG versus DRG) and the fundamentally distinct nerve injury models employed (chronic constriction of the unilateral infraorbital nerve versus spared nerve injury of the sciatic nerve). The TG and DRG have unique functional and anatomical features. The TG is primarily responsible for sensory innervation of the face and oral cavity, while the DRG innervates the limbs and trunk. These structural and functional differences may lead to distinct responses to nerve injury, including variations in TRPC4 expression patterns. In fact, our previous research has already shown differences in the mRNA levels of TRPC channels between the TG and DRG, further suggesting that TRPC channels may serve specific functions in different regions [34]. In addition, chronic constriction of the unilateral infraorbital nerve (CION) and spared nerve injury (SNI) are two distinct nerve injury models that may elicit different cellular and molecular responses. CION specifically targets a branch of the trigeminal nerve for ligation, while SNI involves the ligation of two of the three terminal branches of the sciatic nerve (the common peroneal nerve and the tibial nerve). The differing injury mechanisms and the resulting neuropathic pain phenotypes may influence TRPC4 expression in unique ways. To more comprehensively explore these differences, future studies should investigate the expression of TRPC4 in the TG and DRG following various types of nerve injuries, including CION and SNI. Such research will contribute to a deeper understanding of the underlying mechanisms of TRPC4 expression changes after nerve injury and may potentially reveal new therapeutic targets for neuropathic pain.

Currently, the management of trigeminal neuropathic pain (TNP) in clinical practice settings adopts a comparable strategy to that used for other types of neuropathic pain, with medications for neuropathic pain being the first choice. These treatments unfortunately have a significant failure rate [1]. Hence, it is crucial to ascertain novel therapeutic targets and formulate safer and more efficacious treatments to cater to the unfulfilled medical requirements of TNP [56]. TRPC4, a pharmacological target with great potential, has already seen the development of multiple pharmacological inhibitors, namely Pico145, HC‐070, and ML204. Its crucial role in the research of various diseases such as epilepsy, migraine, severe pulmonary arterial hypertension, and vascular inflammation in arthritis has been demonstrated [5, 57]. Current research indicates that ML204, a specific inhibitor of TRPC4, holds promise as an innovative therapeutic approach for treating TNP. Given the lack of specificity of existing TRPC4 inhibitors and activators, which may also affect TRPC5, we anticipate that with the deepening of TRPC4 structural research, specific small‐molecule drugs targeting TRPC4 will be developed in the future, providing new options for the treatment of trigeminal neuropathic pain. Targeting TRPC4 and its subsequent signaling pathways in the TG offers a novel strategy for treating TNP.

## 5 Conclusion

This study presents the initial evidence indicating that TRPC4 contributes to CION‐induced trigeminal neuropathic pain by facilitating the activation of the downstream transcription factor ATF2 via the Ca^2+^‐ERK/P38 pathway. Therefore, targeting the TRPC4‐ERK/P38‐ATF2 pathway in the trigeminal ganglion (TG) could offer a novel therapeutic strategy for managing trigeminal neuropathic pain.

## Author Contributions

G.C., Y.C., X.Z., and Y.Z.: conceptualization. X.K. and H.C.: formal analysis. X.K.: investigation. X.K., H.C., F.L., X.Z., and Q.H.: data curation. X.K., H.C., and Y.C.: manuscript editing. X.K., H.C., and Y.C.: writing – original draft preparation. X.Z., Y.W. and Y.C.: writing – review and editing. Y.C. and G.C.: supervision. Y.C. and G.C.: project administration. Y.Z., Y.C., Y.C. and G.C.: funding acquisition. All authors read and approved the final manuscript.

## Conflicts of Interest

The authors declare no conflicts of interest.

## Supporting information


**Figure S1.** (A) At the end of the experiment, post‐validation was performed on the virus injection sites (TG). Representative immunofluorescence images showing the colocalization of GFP and TRPC4 in the TG in the CION + Vehicle and CION + ShRNA groups. (B) At the end of the experiment, real‐time fluorescent quantitative PCR was employed to determine the expression levels of *Trpc1*‐*Trpc7* mRNA in the trigeminal ganglia (TG) of CION mice in both the scramble virus injection group and the *Trpc4* ShRNA injection group, with normalization to *Gapdh*. (***p < 0.001, Multiple unpaired Mann–Whitney tests. *n* = 3 mice/group).

**Figure S2.** Control plasmid and TRPC4 plasmid map.

**Figure S3.** In female mice, chronic constriction of the unilateral infraorbital nerve (CION) induced trigeminal neuropathic pain and was accompanied by an upregulation of TRPC4 expression in the trigeminal ganglion. (A–C) In C57BL/6 female mice, constriction of the infraorbital nerve elicits mechanical allodynia (A), non‐evoked nociceptive behavior (B), and cold allodynia (C), persisting from day 3 until day 28 post‐surgery (*p < 0.05, **p < 0.01, and ***p < 0.001 versus sham, Two‐way repeated measured ANOVA, followed by Tukey’s multiple comparisons test.). (D) The expression of TRPC4 protein in the ipsilateral trigeminal ganglion (TG) was assessed after CION or sham surgery, with normalization to GAPDH. (***p < 0.001 vs. sham, Student’s *t*‐test; *n* = 6 mice/group).

**Figure S4.** Double‐stained immunofluorescent images showing the co‐localization of TRPC4 with NeuN (A), GS (B), NF200 (C), CGRP (D), IB4 (E).

**Figure S5.** The Kyoto Encyclopedia of Genes and Genomes (KEGG) pathway enrichment analysis of differentially expressed genes in mouse model of trigeminal neuropathic pain. Pathways such as PI3K‐Akt signaling and MAPK signaling prominently enriched, highlighting their roles in the biological processes in mouse model of trigeminal neuropathic pain.

**Figure S6.** The injection of Englerin A into TG and CION does not activate PI3K‐Akt and JNK. (A, B) Western blot analysis of p‐mTOR, p‐PI3K, p‐Akt, and p‐JNK protein levels in the trigeminal ganglion (TG) of sham and CION mice groups, normalized to GAPDH. (multiple unpaired Mann–Whitney tests; *n* = 6 mice/group). (C, D) Western blot analysis was performed to assess the protein levels of p‐mTOR, p‐PI3K, p‐Akt, and p‐JNK in the trigeminal ganglion (TG) of Vehicle‐treated and Englerin A‐treated mice groups, normalized to GAPDH. (multiple unpaired Mann–Whitney tests; *n* = 6 mice/group).

**Table S1.** Quantitative real‐time RT‐PCR primer sequence.

**Data S1.** Xxxxxx.

## Data Availability

The data that support the findings of this study are available from the corresponding author upon reasonable request.
