# Supplementary figures and images for: TRPC4 Mediates Trigeminal Neuropathic Pain via Ca2+‐ERK/P38‐ATF2 Pathway in the Trigeminal Ganglion of Mice
Source: CNS Neurosci Ther. 2025 Apr 9;31(4):e70368. doi: 10.1111/cns.70368 (PMC11979714; doi:10.1111/cns.70368)

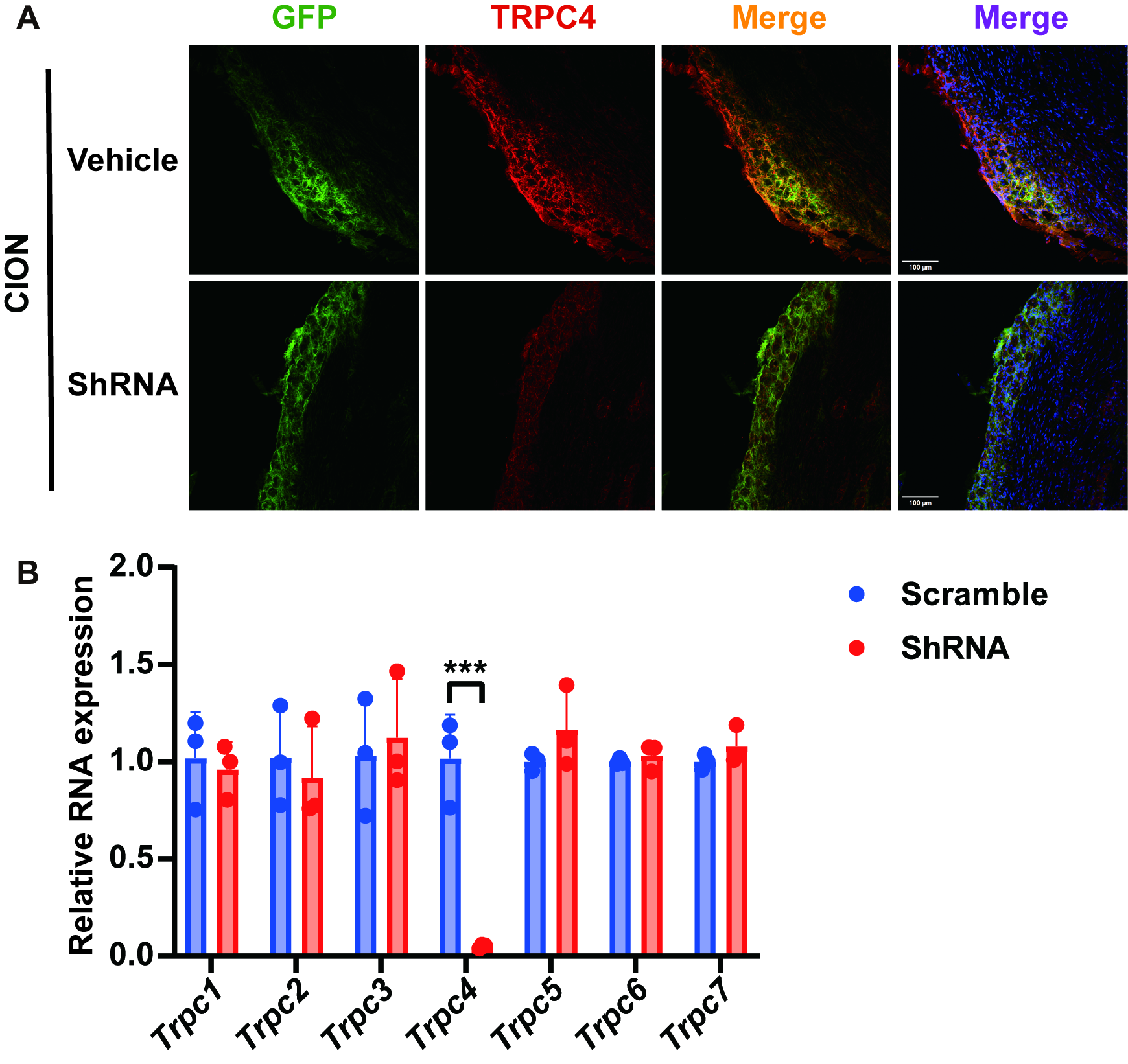

Supplement: Supplementary file 1 — Figure S1. (A) At the end of the experiment, post‐validation was performed on the virus injection sites (TG). Representative immunofluorescence images showing the colocalization of GFP and TRPC4 in the TG in the CION + Vehicle and CION + ShRNA groups. (B) At the end of the experiment, real‐time fluorescent quantitative PCR was employed to determine the expression levels of Trpc1‐Trpc7 mRNA in the trigeminal ganglia (TG) of CION mice in both the scramble virus injection group and the Trpc4 ShRNA injection group, with normalization to Gapdh. (***p < 0.001, Multiple unpaired Mann–Whitney tests. n = 3 mice/group). [file CNS-31-e70368-s004.tif]

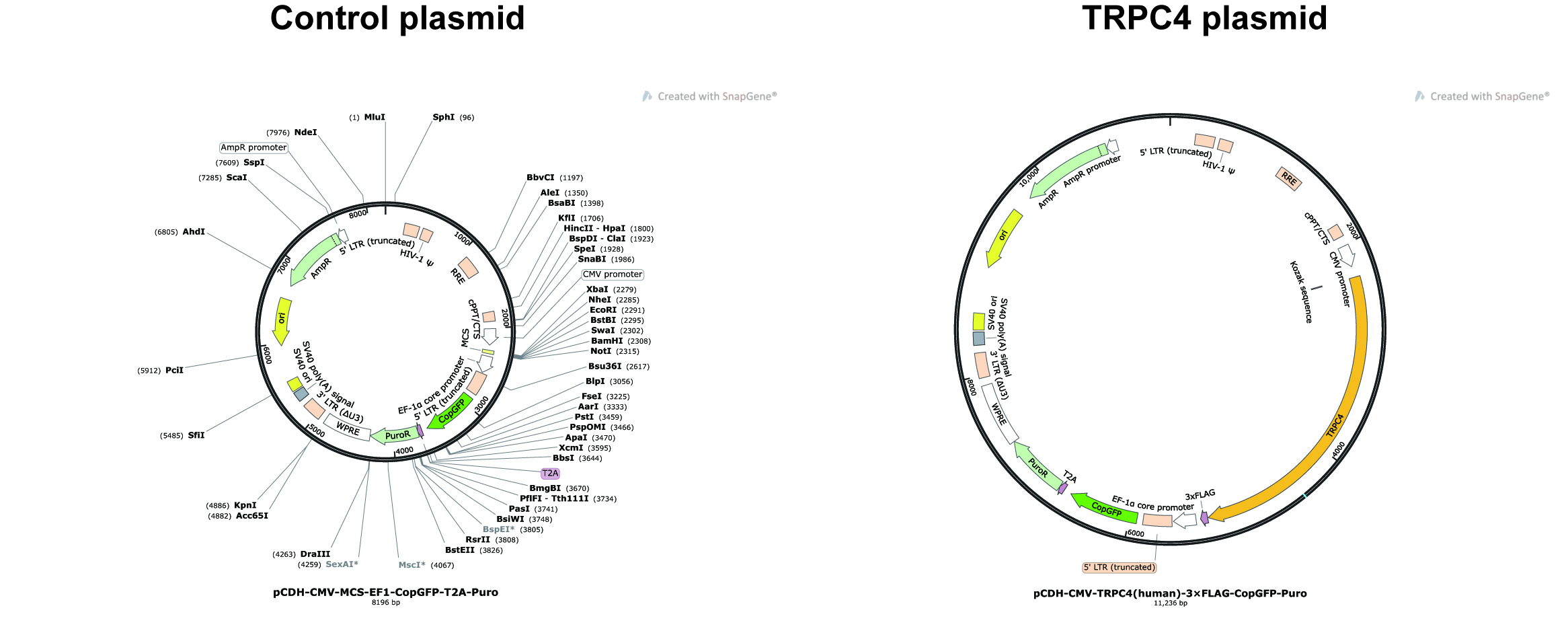

Supplement: Supplementary file 2 — Figure S2. Control plasmid and TRPC4 plasmid map. [file CNS-31-e70368-s007.tif]

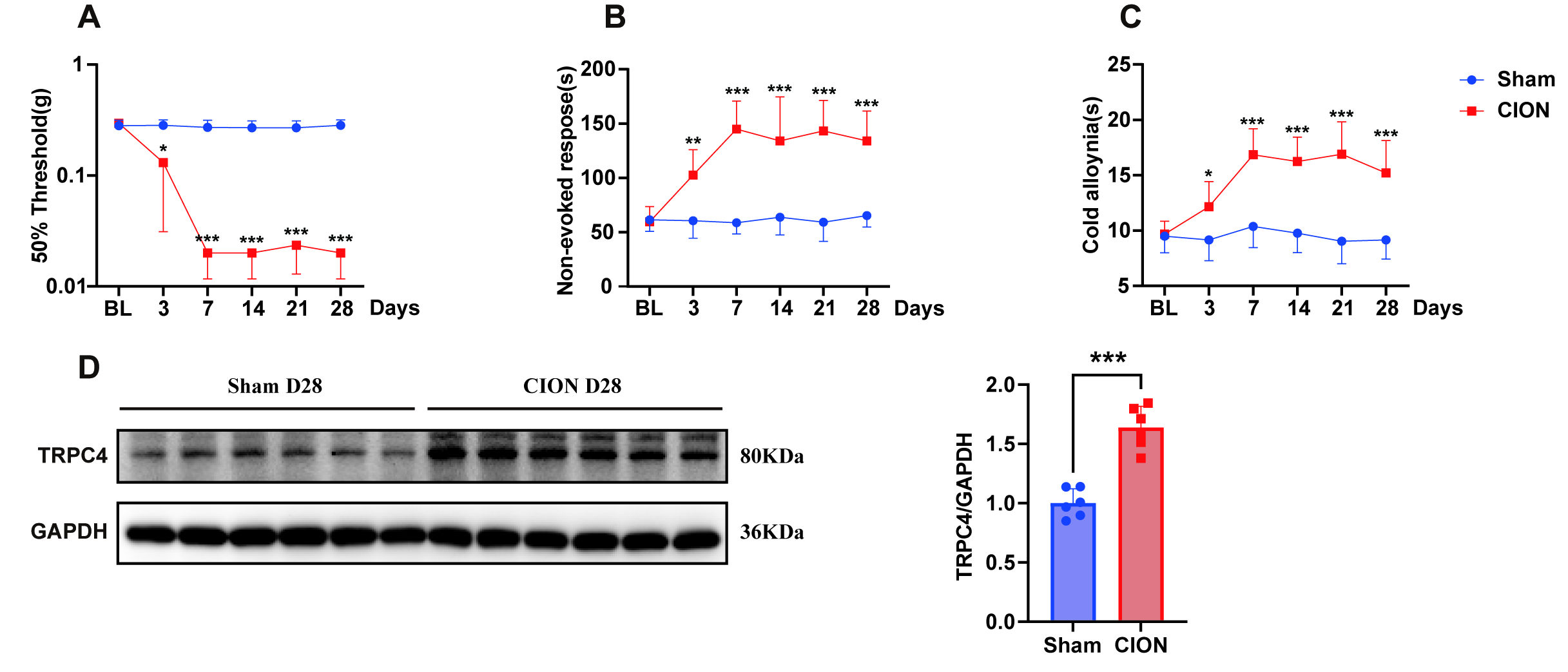

Supplement: Supplementary file 3 — Figure S3. In female mice, chronic constriction of the unilateral infraorbital nerve (CION) induced trigeminal neuropathic pain and was accompanied by an upregulation of TRPC4 expression in the trigeminal ganglion. (A–C) In C57BL/6 female mice, constriction of the infraorbital nerve elicits mechanical allodynia (A), non‐evoked nociceptive behavior (B), and cold allodynia (C), persisting from day 3 until day 28 post‐surgery (*p < 0.05, **p < 0.01, and ***p < 0.001 versus sham, Two‐way repeated measured ANOVA, followed by Tukey’s multiple comparisons test.). (D) The expression of TRPC4 protein in the ipsilateral trigeminal ganglion (TG) was assessed after CION or sham surgery, with normalization to GAPDH. (***p < 0.001 vs. sham, Student’s t‐test; n = 6 mice/group). [file CNS-31-e70368-s005.tif]

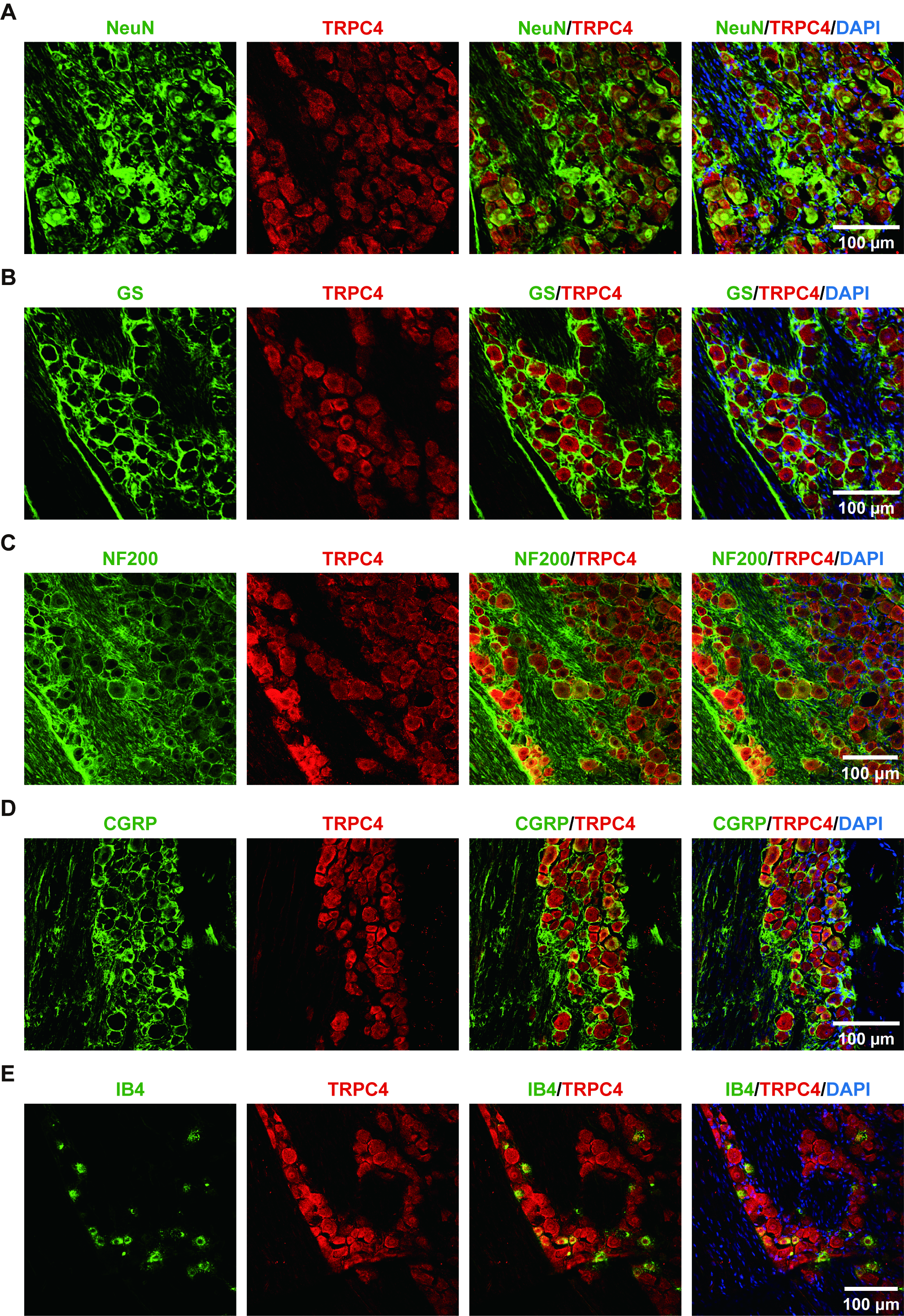

Supplement: Supplementary file 4 — Figure S4. Double‐stained immunofluorescent images showing the co‐localization of TRPC4 with NeuN (A), GS (B), NF200 (C), CGRP (D), IB4 (E). [file CNS-31-e70368-s006.tif]

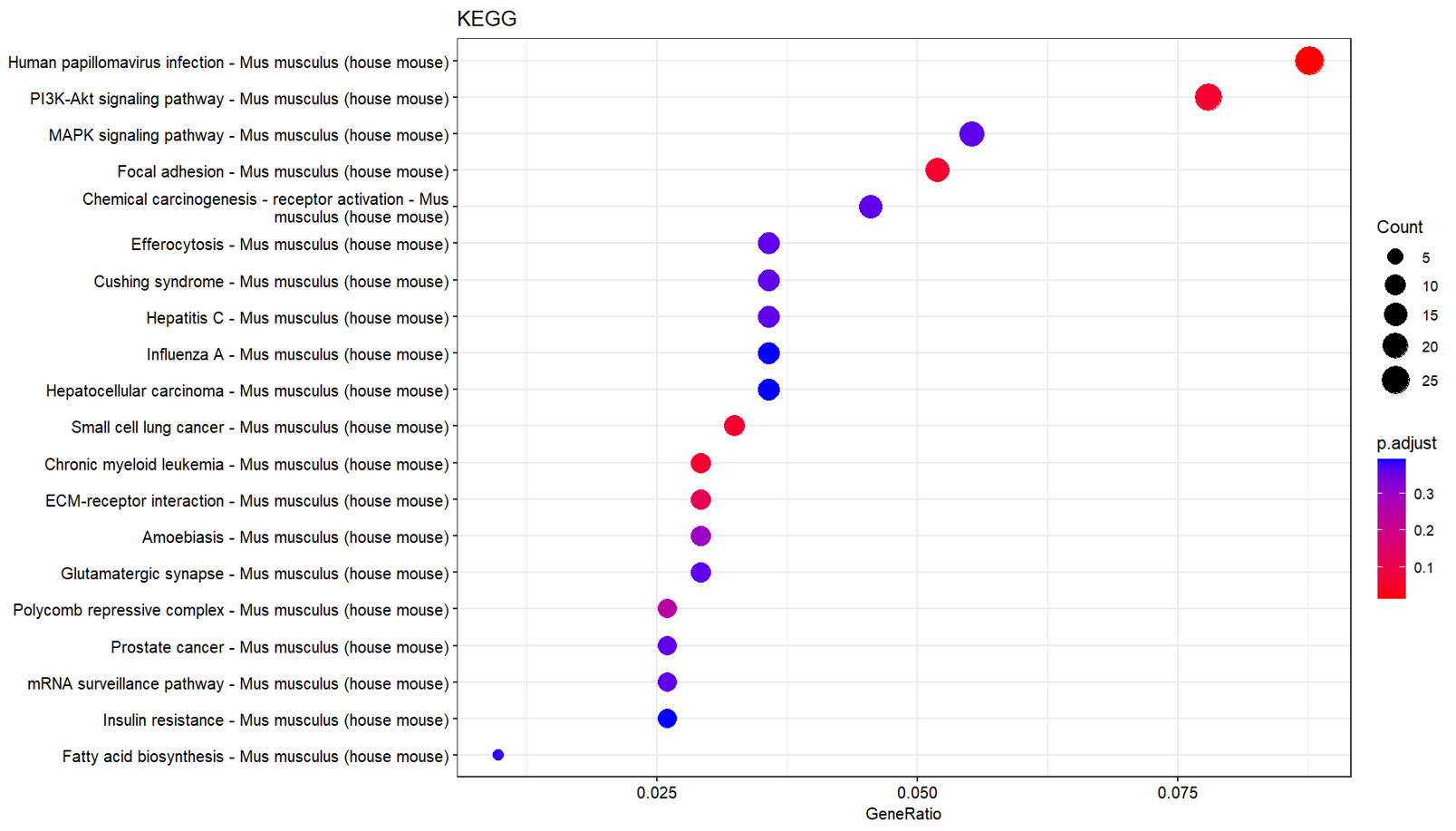

Supplement: Supplementary file 5 — Figure S5. The Kyoto Encyclopedia of Genes and Genomes (KEGG) pathway enrichment analysis of differentially expressed genes in mouse model of trigeminal neuropathic pain. Pathways such as PI3K‐Akt signaling and MAPK signaling prominently enriched, highlighting their roles in the biological processes in mouse model of trigeminal neuropathic pain. [file CNS-31-e70368-s001.tif]

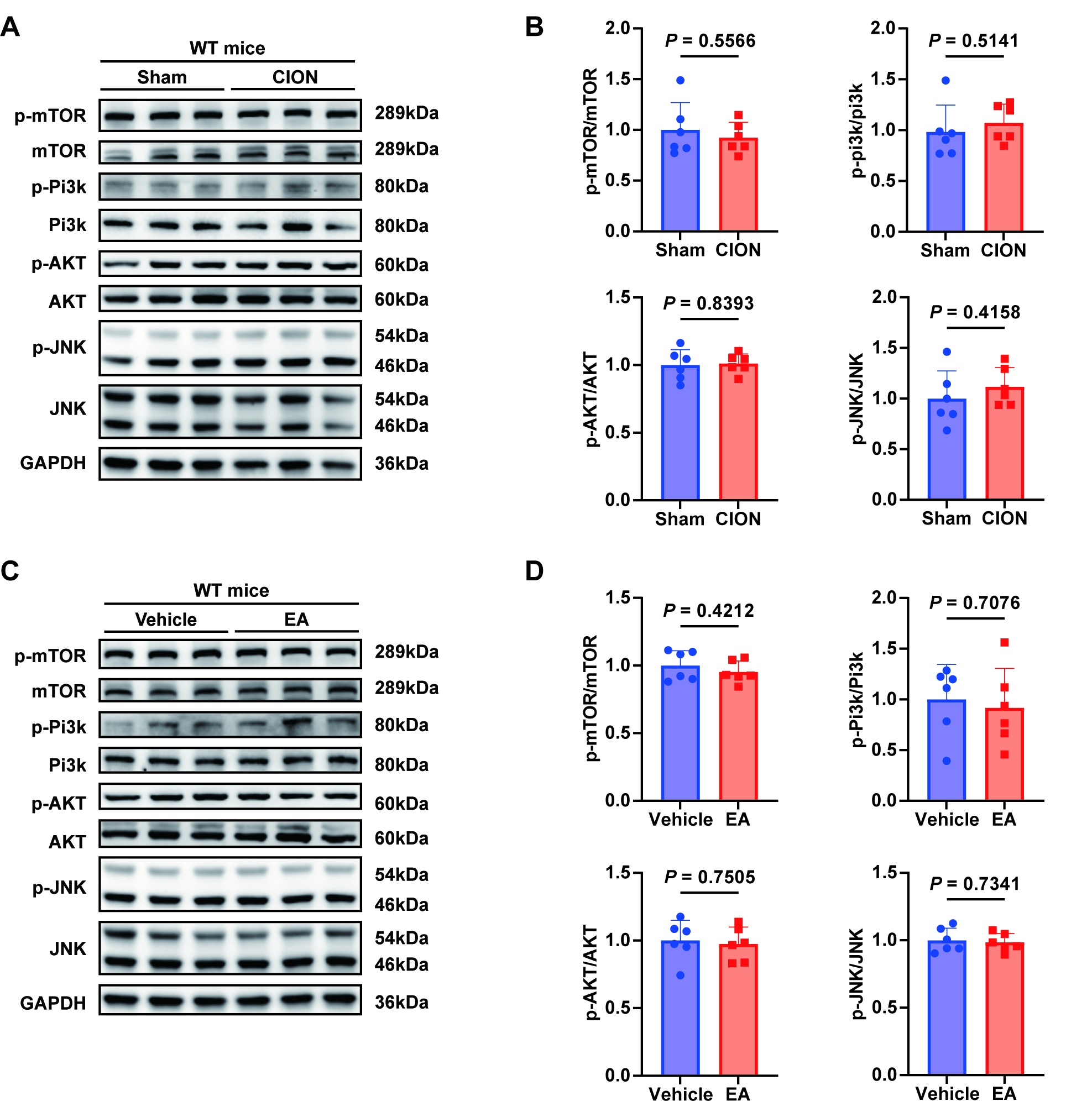

Supplement: Supplementary file 6 — Figure S6. The injection of Englerin A into TG and CION does not activate PI3K‐Akt and JNK. (A, B) Western blot analysis of p‐mTOR, p‐PI3K, p‐Akt, and p‐JNK protein levels in the trigeminal ganglion (TG) of sham and CION mice groups, normalized to GAPDH. (multiple unpaired Mann–Whitney tests; n = 6 mice/group). (C, D) Western blot analysis was performed to assess the protein levels of p‐mTOR, p‐PI3K, p‐Akt, and p‐JNK in the trigeminal ganglion (TG) of Vehicle‐treated and Englerin A‐treated mice groups, normalized to GAPDH. (multiple unpaired Mann–Whitney tests; n = 6 mice/group). [file CNS-31-e70368-s002.tif]
