# Supplementary material for: TRPC4 Mediates Trigeminal Neuropathic Pain via Ca2+‐ERK/P38‐ATF2 Pathway in the Trigeminal Ganglion of Mice
Source: CNS Neurosci Ther. 2025 Apr 9;31(4):e70368. doi: 10.1111/cns.70368 (PMC11979714; doi:10.1111/cns.70368)
Supplement: Supplementary file 7 — Table S1. Quantitative real‐time RT‐PCR primer sequence. [file CNS-31-e70368-s008.docx]

**Supplementary Material**

**Table**

**Supplementary Table 1:** Quantitative Real-Time RT-PCR Primer Sequence

| **Gene** | **Forward Primer (5’-3’)** | **Reverse Primer (5’-3’)** | **Accession Number** | |
| --- | --- | --- | --- | --- |
| *Trpc1* | TACGGTTGTCAGTCCGCAGA | TCGTTTTGGCCGATGATTAAGTA | NM_011643.4 | |
| *Trpc2* | GCCATGTGGTGTCATTTTCCT | GTTGTCCAGTCTTGTCCTGAG | NM_011644.3 | |
| *Trpc3* | TCGAGAGGCCACACGACTA | CTGGACAGCGACAAGTATGC | NM_019510.2 | |
| *Trpc4* | TGTATCTGGCAACAATCTCCTTG | CATGTCCCATGATTCCCGTGG | NM_016984.3 | |
| *Trpc5* | GTGTATCCAGTTCGGAGGTAGA | CCTCGCTTGATAAGGCAATGA | NM_009428.3 | |
| *Trpc6* | AGCCAGGACTATTTGCTGATGG | AACCTTCTTCCCTTCTCACGA | NM_013838.2 | |
| *Trpc7* | CTTCCTGGACTCGGCTGAGTA | GCGTTCTGCCCCATGTAGT | NM_012035.3 | |
| *Gapdh* | AGGTCGGTGTGAACGGATTTG | GGGGTCGTTGATGGCAACA | | NM_001289726.1 |
