# Supplementary material for: TRPC4 Mediates Trigeminal Neuropathic Pain via Ca2+‐ERK/P38‐ATF2 Pathway in the Trigeminal Ganglion of Mice
Source: CNS Neurosci Ther. 2025 Apr 9;31(4):e70368. doi: 10.1111/cns.70368 (PMC11979714; doi:10.1111/cns.70368)

Uncropped western blot images

Full unedited blots for Figure 1I

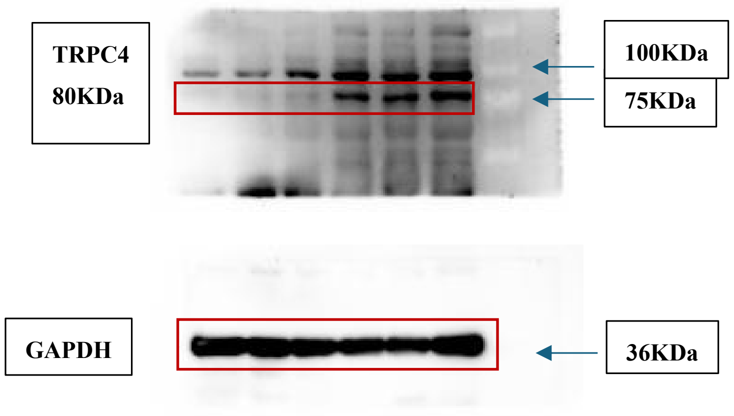

Full unedited blots for Figure 1K

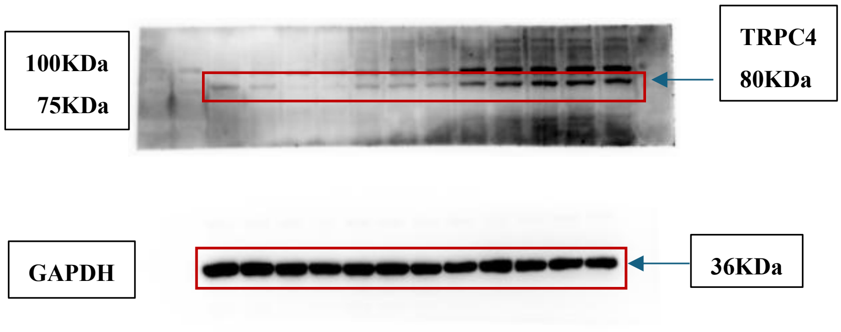

Full unedited blots for Figure 4B

TRPC4

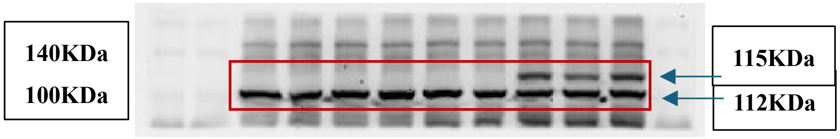

GAPDH

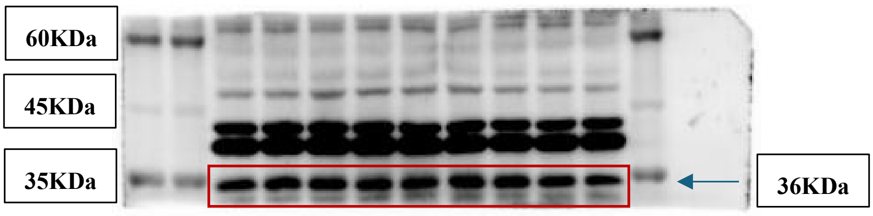

Full unedited blots for Figure 5A

p-mTOR

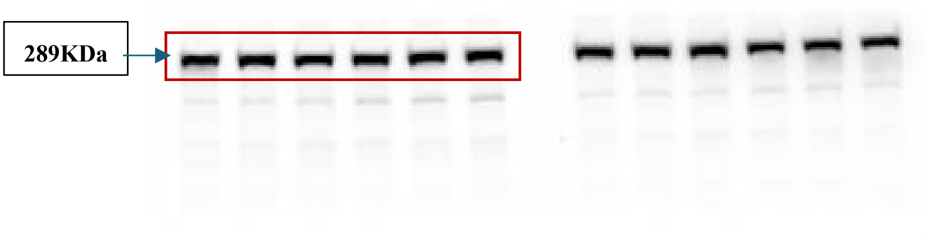

mTOR

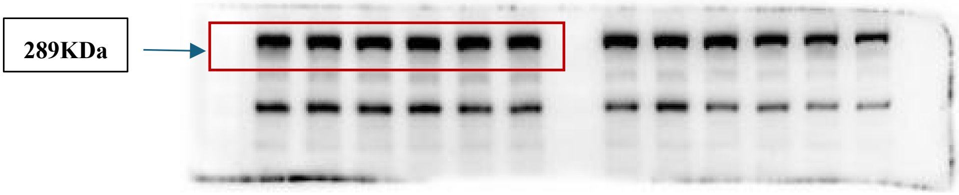

p-PI3K

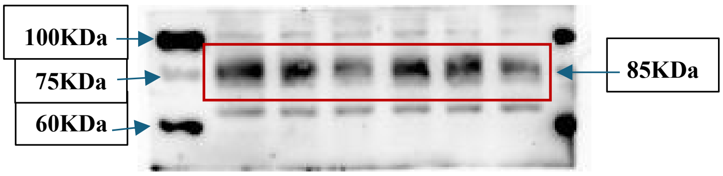

PI3K

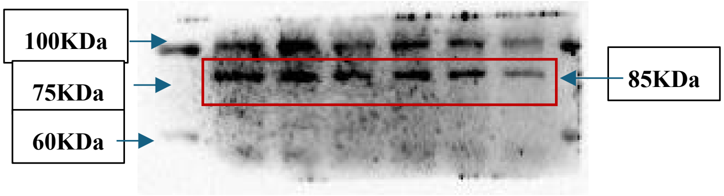

Akt

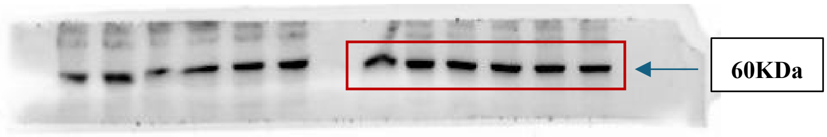

P-Akt

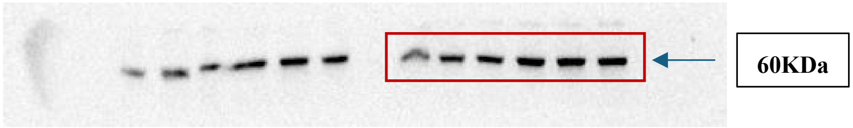

GAPDH

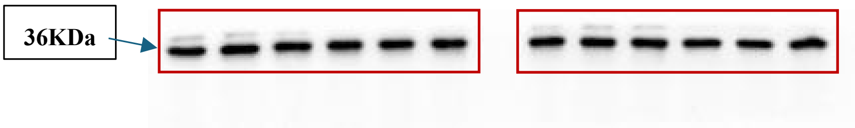

Full unedited blots for Figure 5B

P-ERK

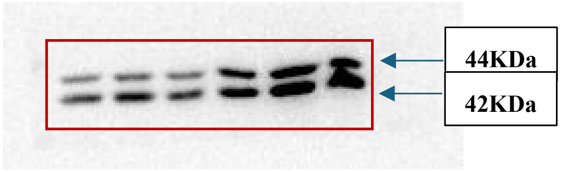

ERK

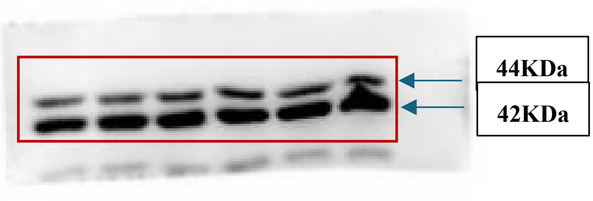

p-P38

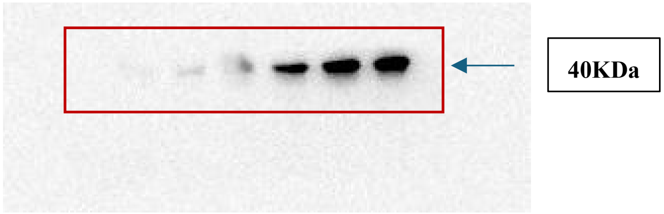

P38

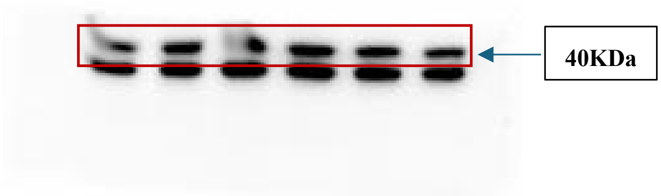

GAPDH

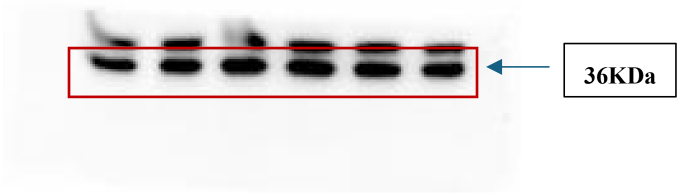

p-JNK

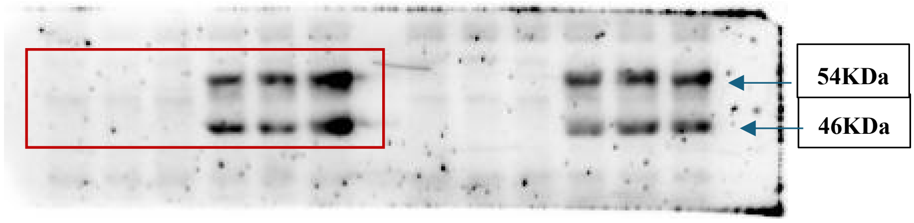

JNK

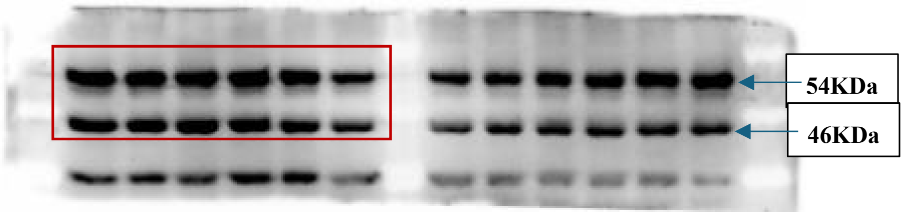

GAPDH

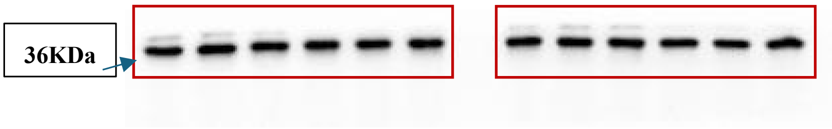

Full unedited blots for Figure 5C

TRPC4

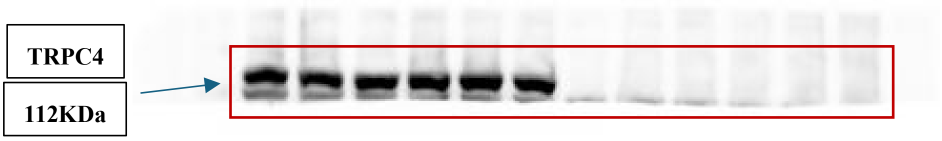

p-Akt

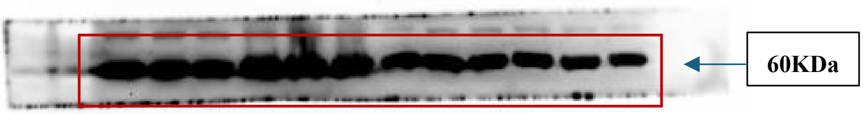

Akt

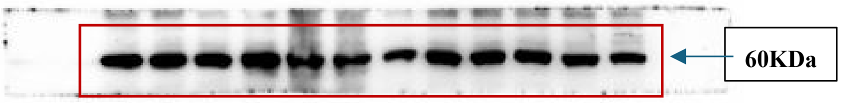

p-P38

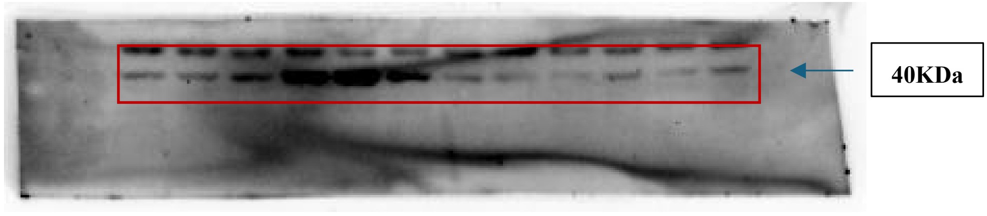

P38

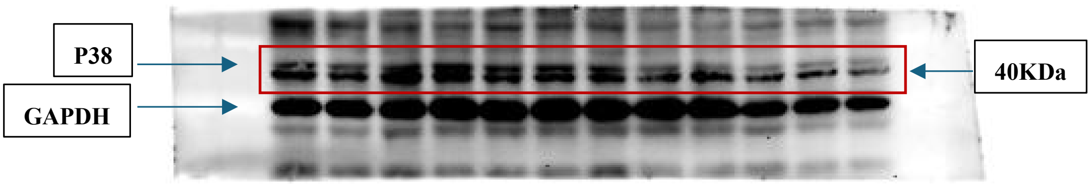

p-ERK

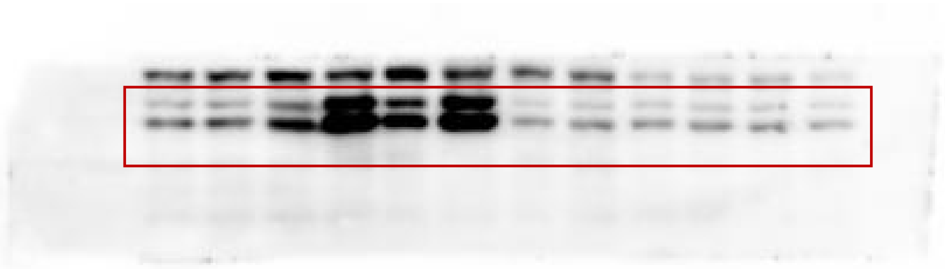

ERK

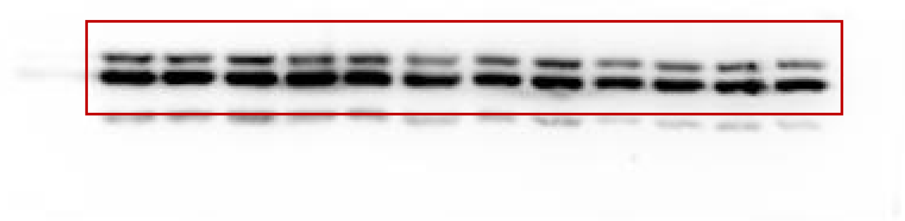

p-JNK

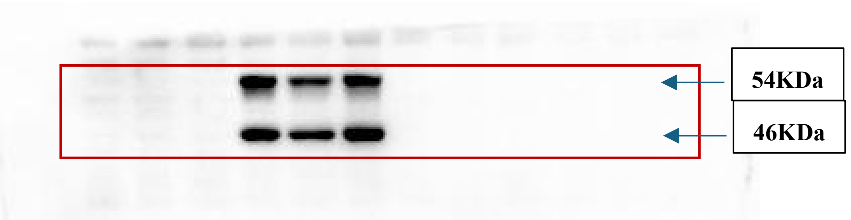

JNK

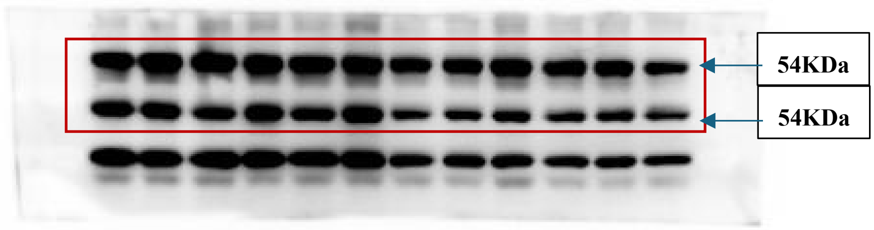

GAPDH

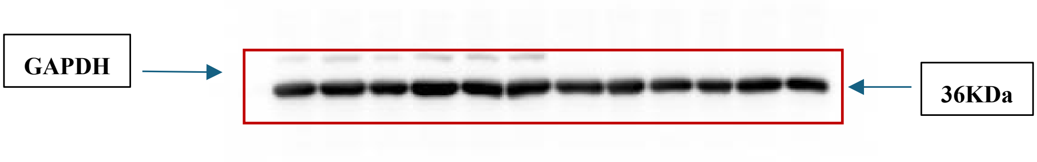

Full unedited blots for Figure 6A

p-mTOR

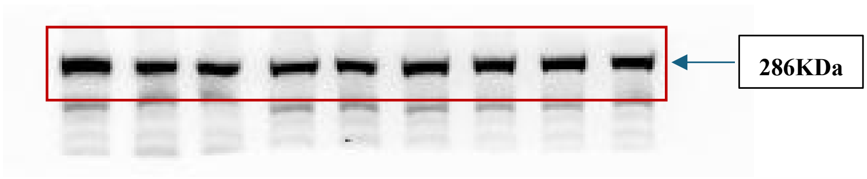

mTOR

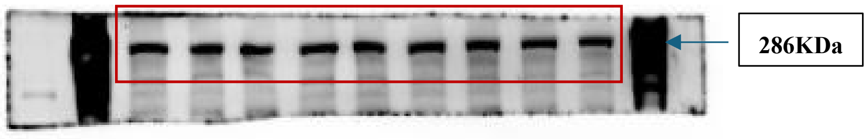

p-PI3K

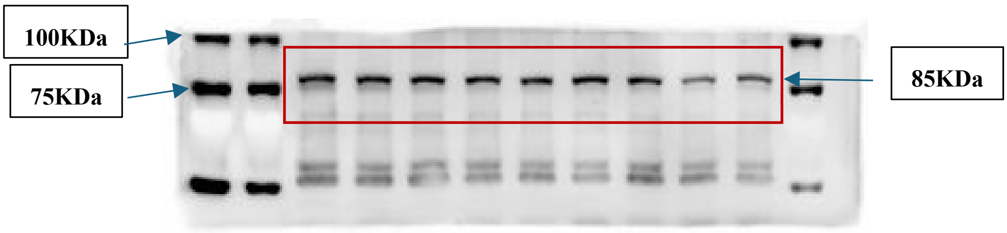

PI3K

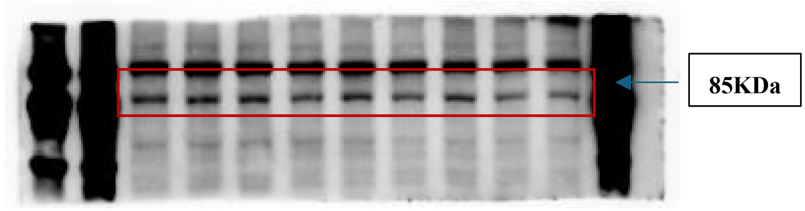

p-Akt

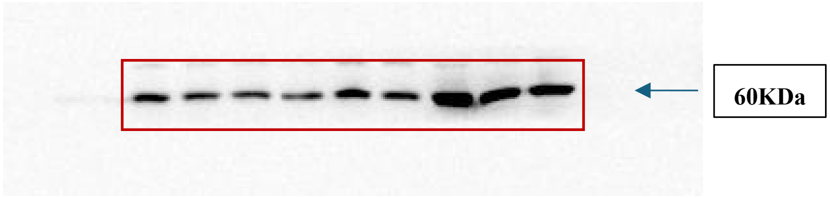

Akt

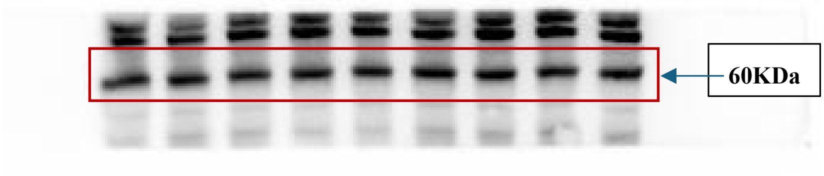

P-ERK

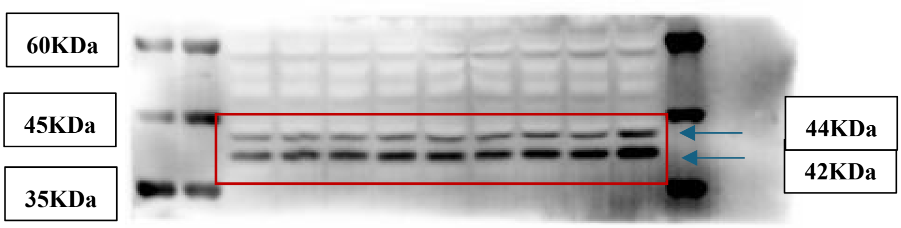

ERK

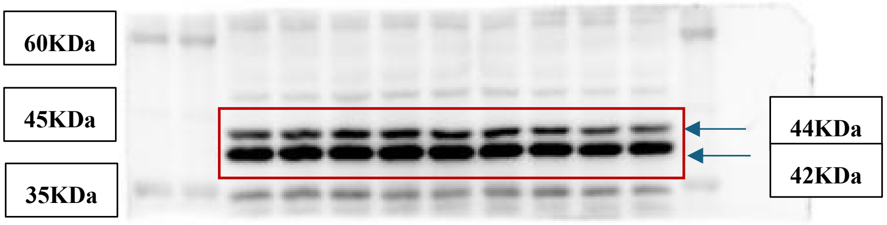

p-P38

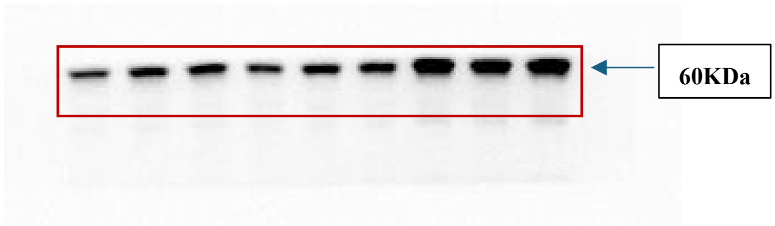

P38

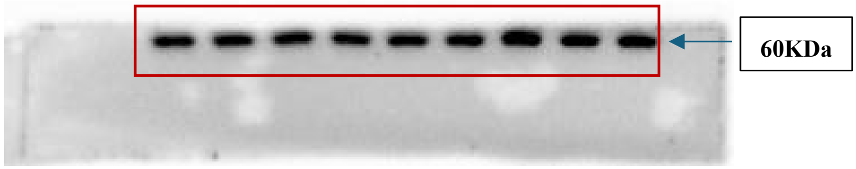

p-JNK

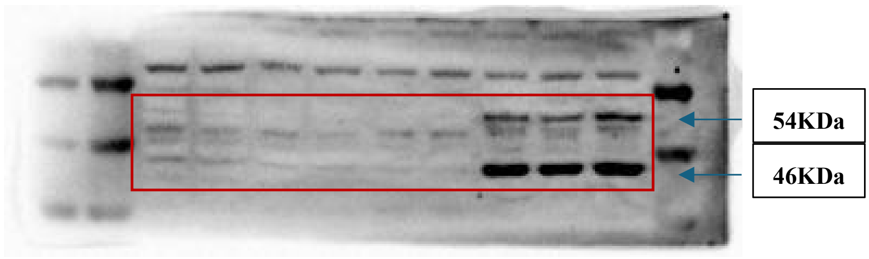

JNK

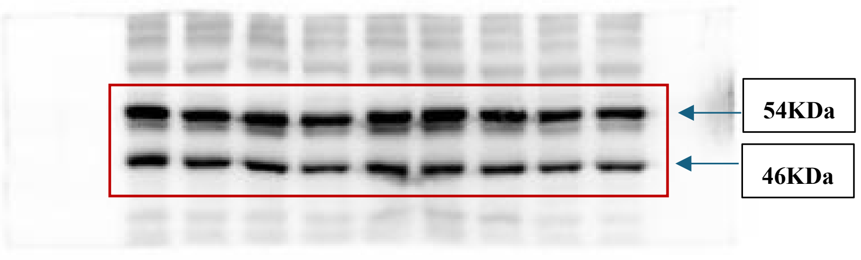

GAPDH

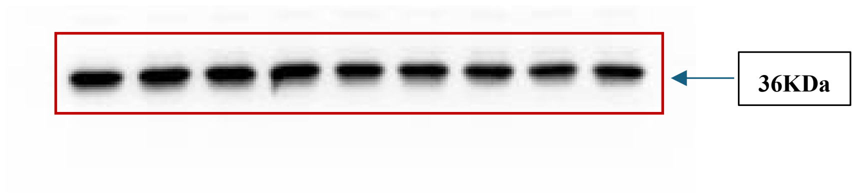

Full unedited blots for Figure 6C

TRPC4

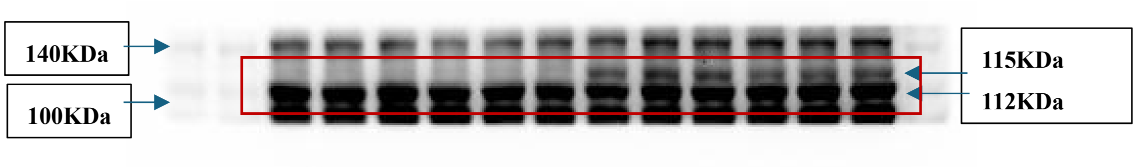

p-AKT

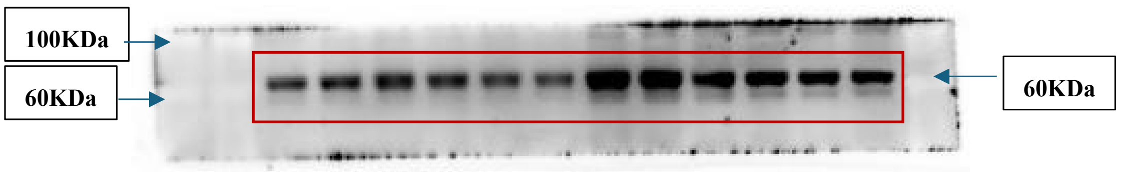

AKT

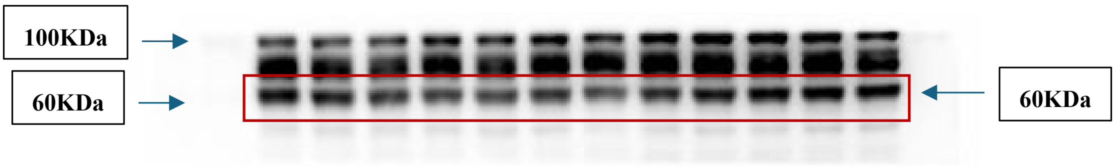

p-ERK

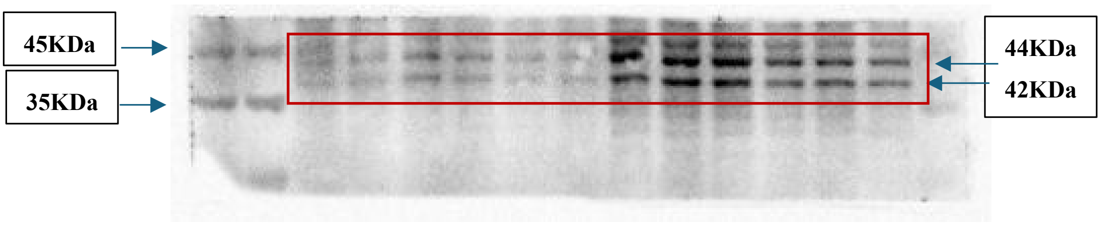

ERK

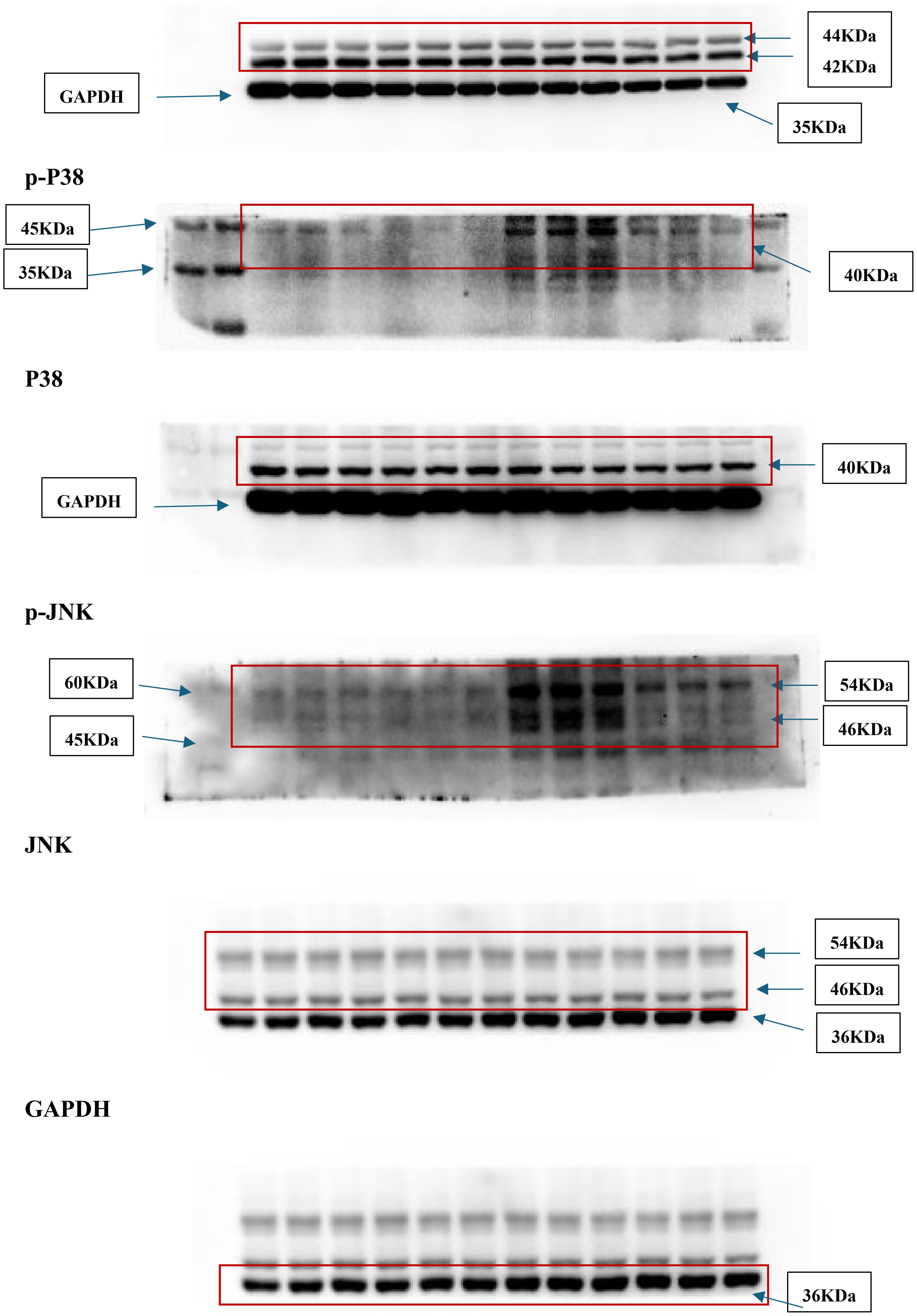

Full unedited blots for Figure 7A

p-ATF2

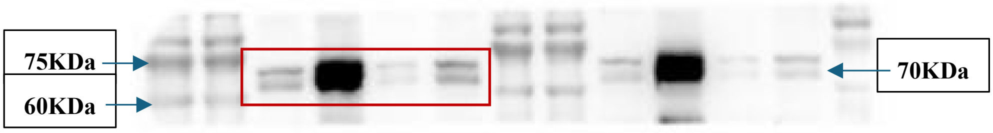

ATF2

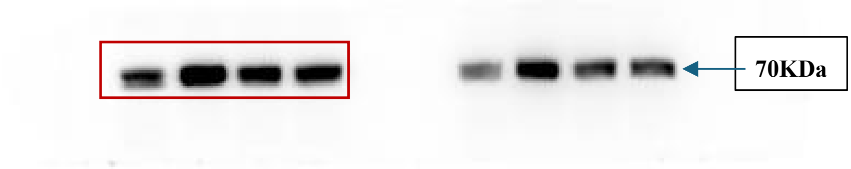

p-P38

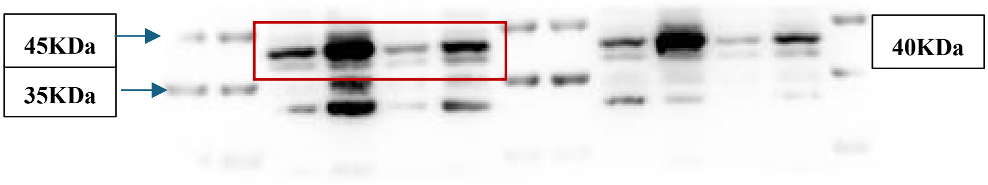

P38

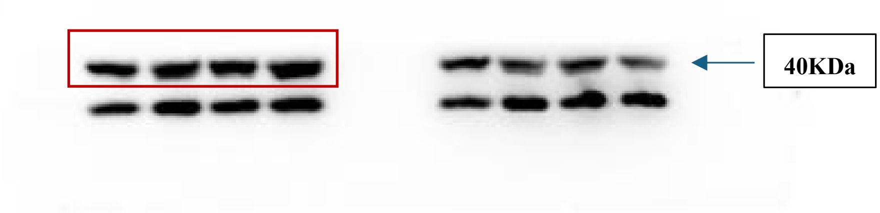

Full unedited blots for Figure 7D

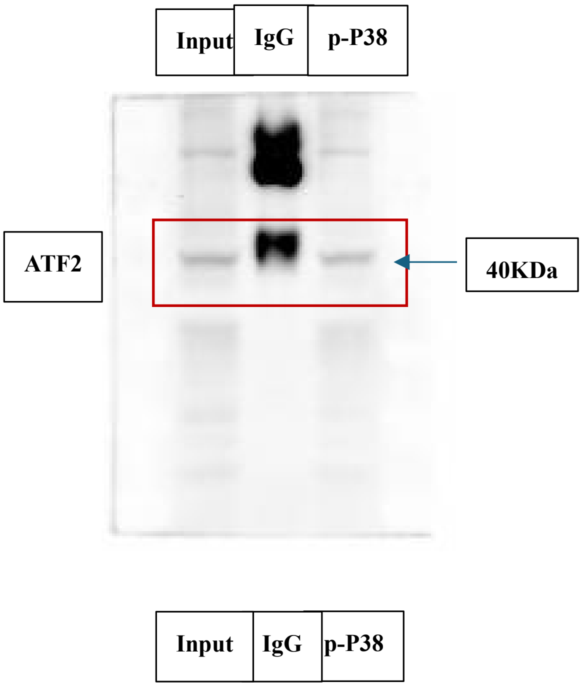

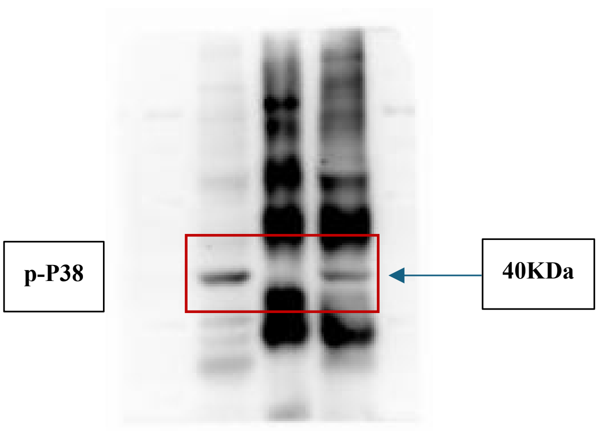

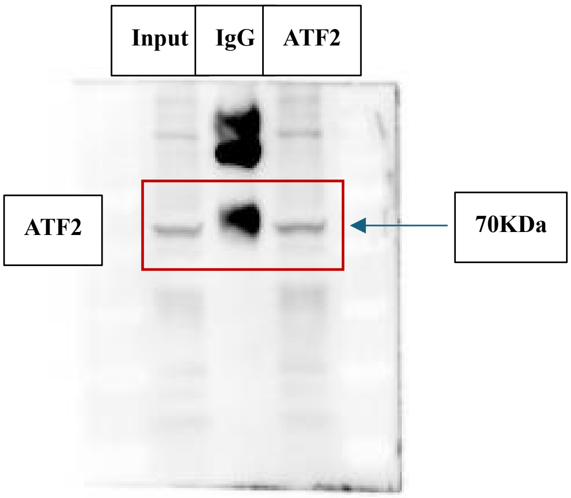

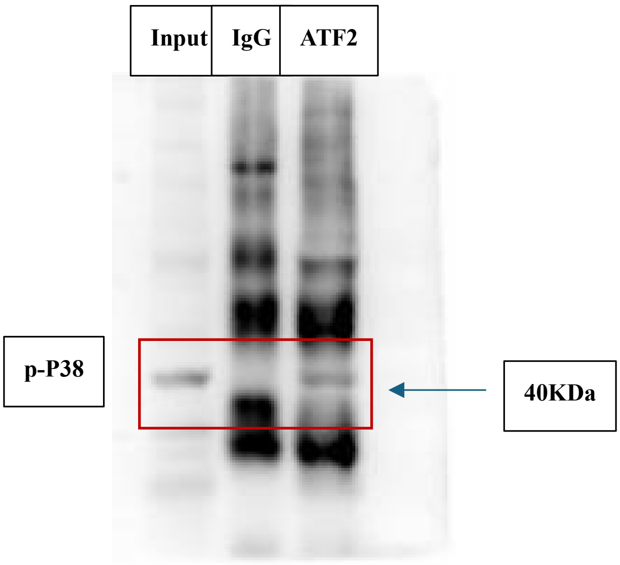

Full unedited blots for Figure 7E

p-ERK

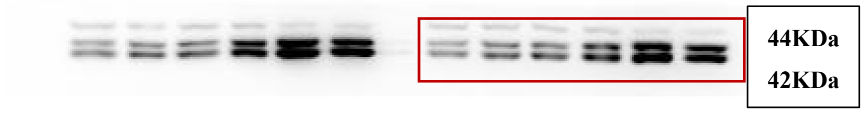

ERK

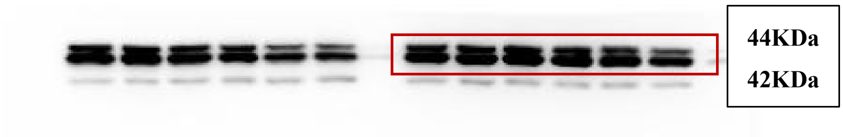

GAPDH

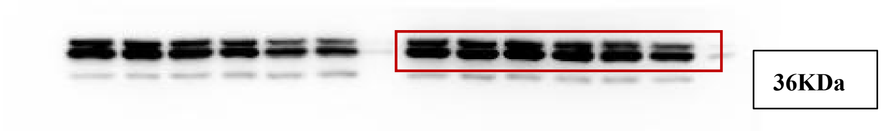

p-P38

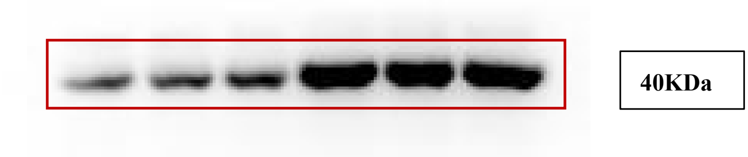

P38

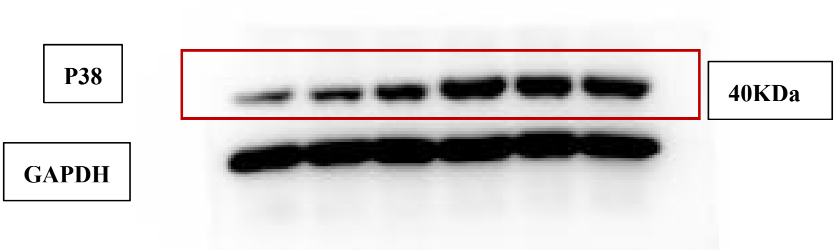

p-ATF2

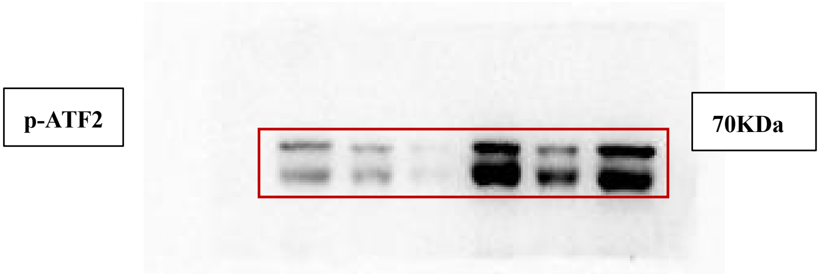

ATF2

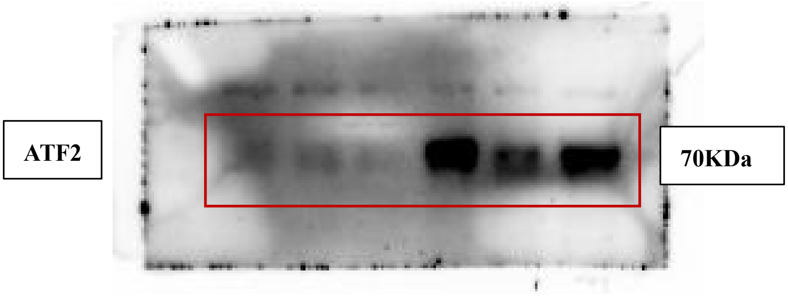

GAPDH

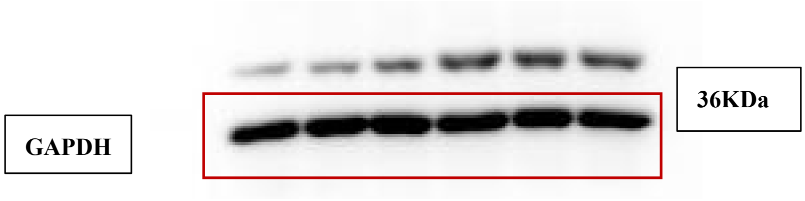

Full unedited blots for Figure 7G

p-ERK

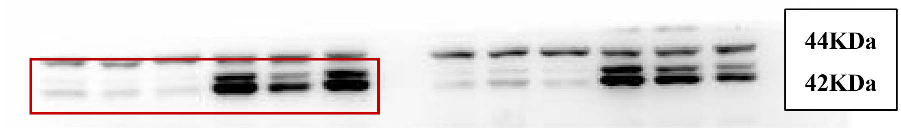

ERK

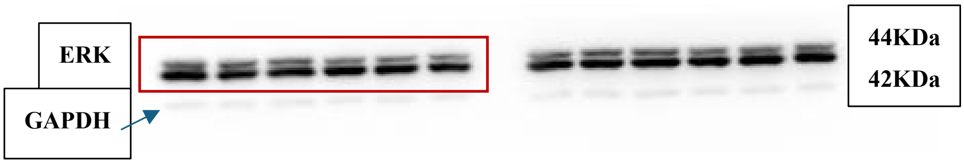

p-P38

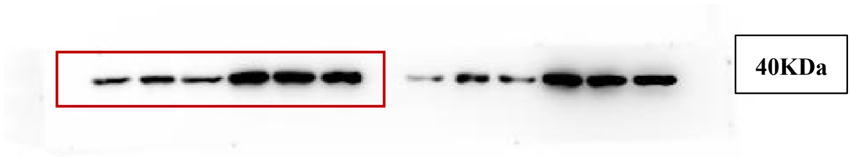

P38

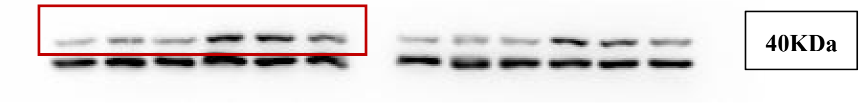

GAPDH

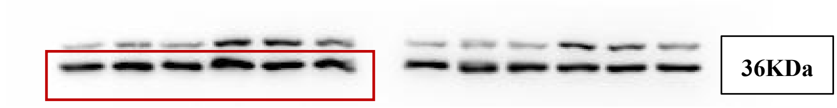

GAPDH

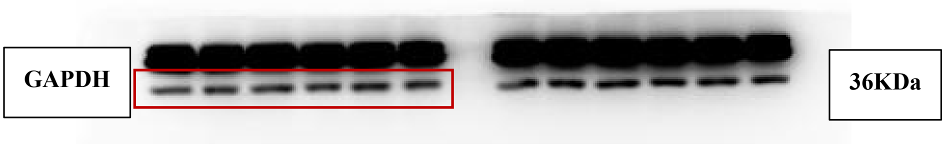

p-ATF2

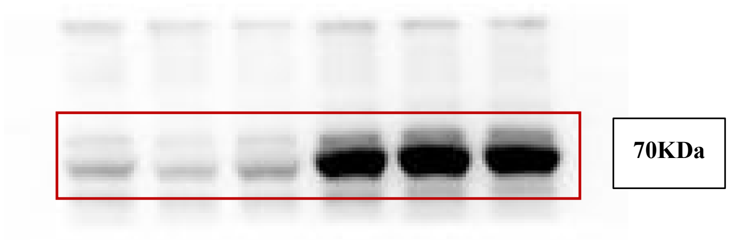

ATF2

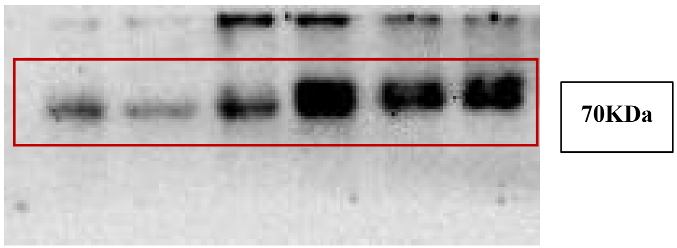

Full unedited blots for Figure 8D

TRPC4

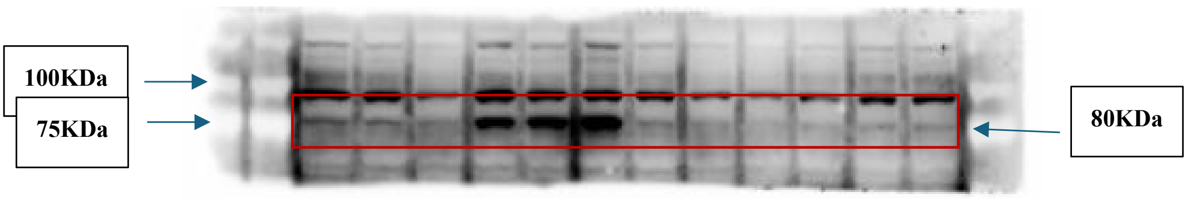

p-P38

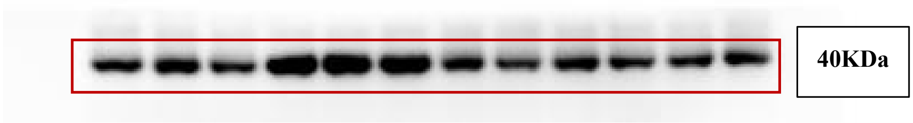

P38

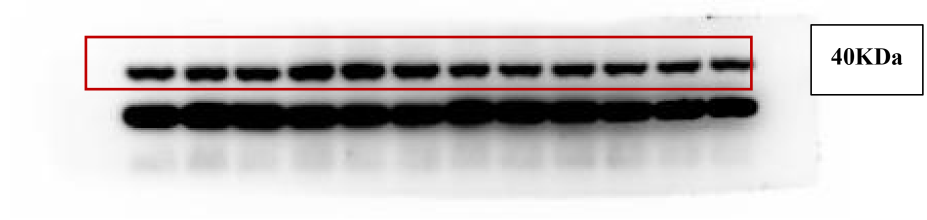

GAPDH

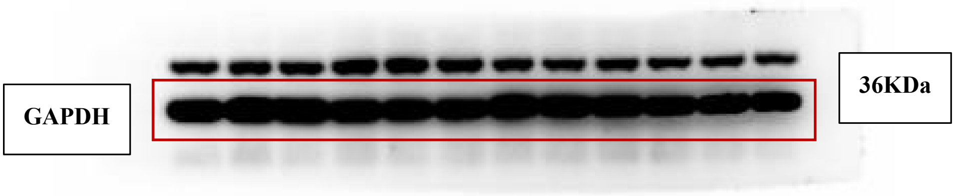

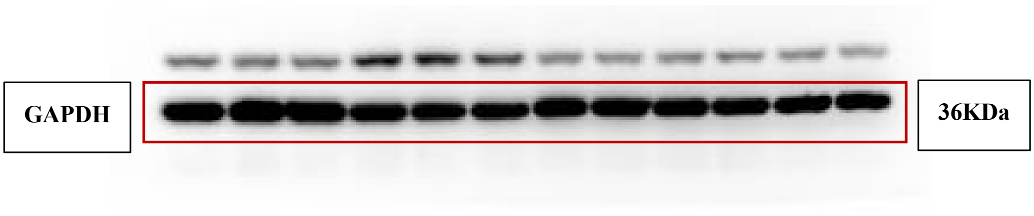

p-ERK

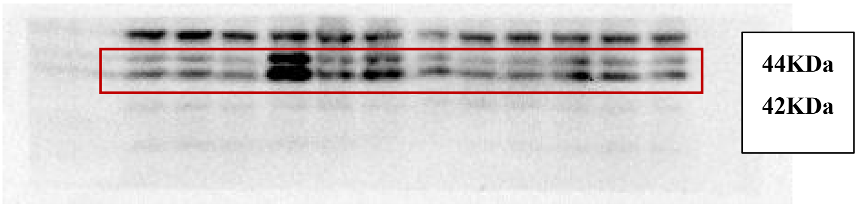

ERK

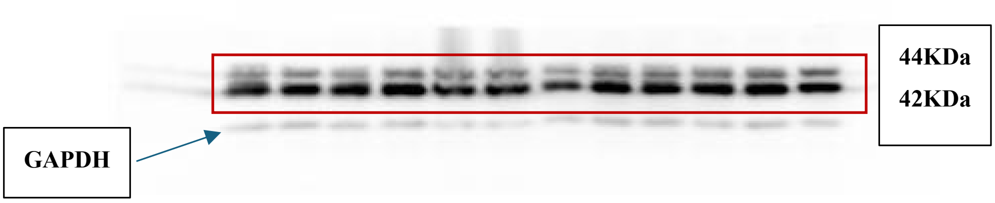

p-ATF2

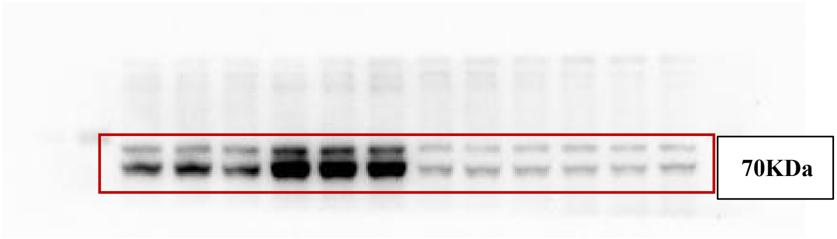

ATF2

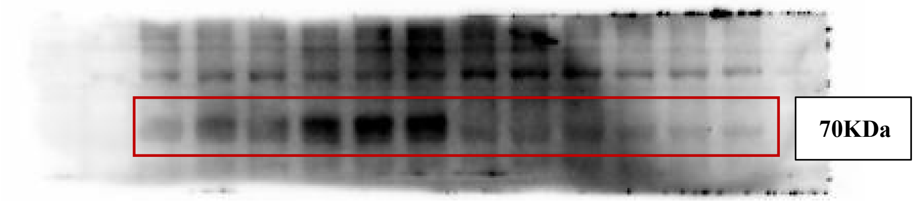

Full unedited blots for Figure 9D

p-ERK

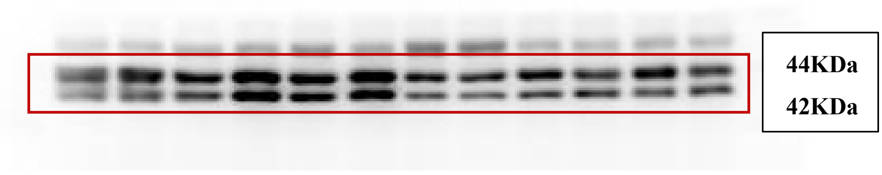

ERK

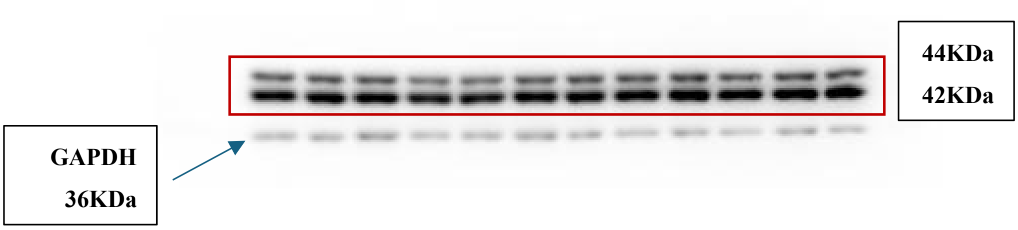

p-P38

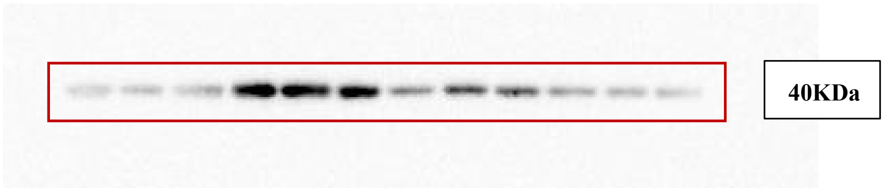

P38

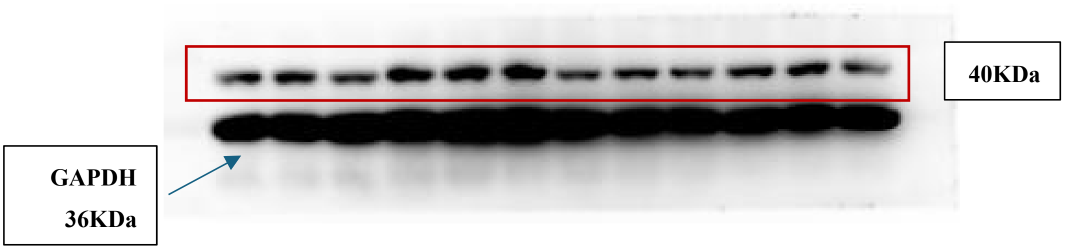

GAPDH

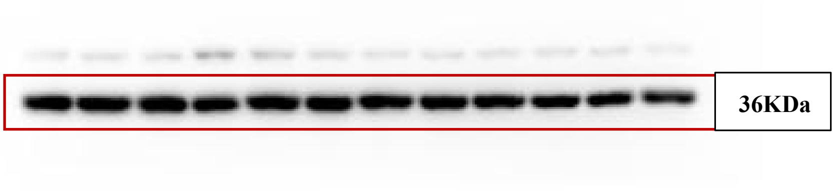

p-ATF2

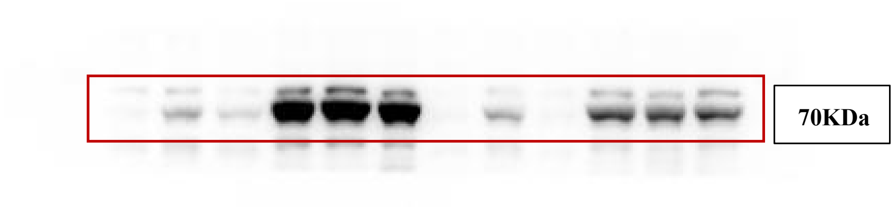

ATF2

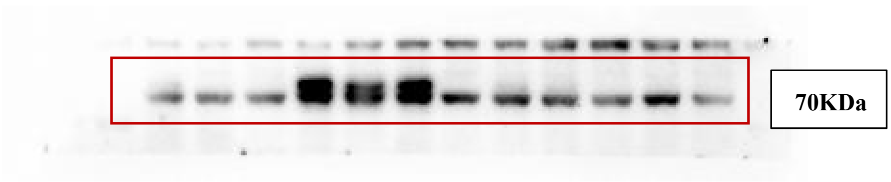

Full unedited blots for Supplementary Figure 3D

TRPC4

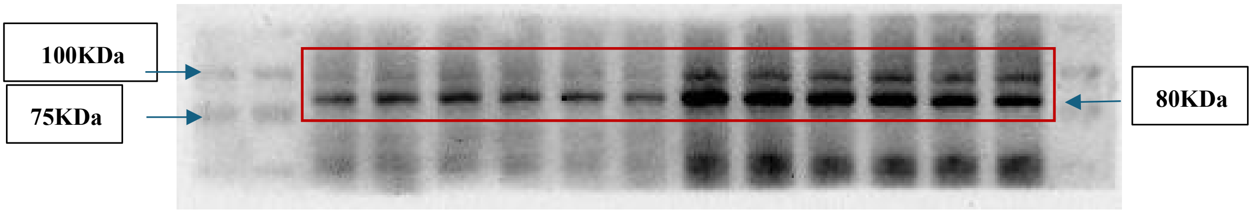

GAPDH

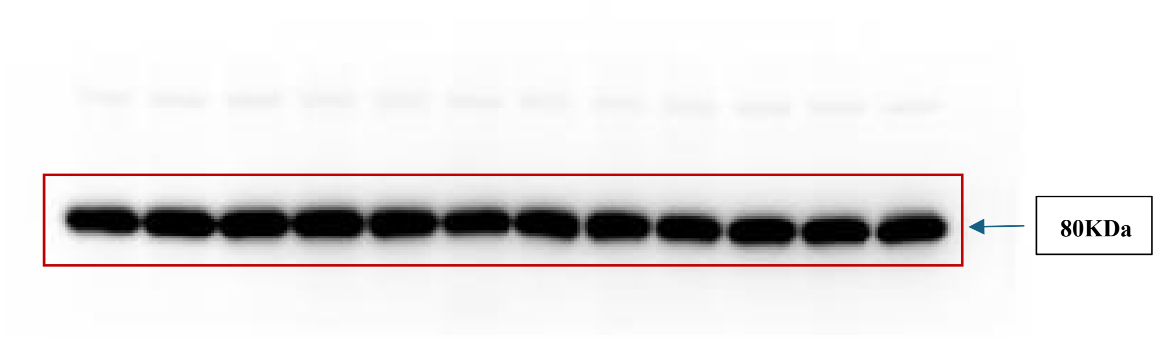

Full unedited blots for Supplementary Figure 6A

p-mTOR

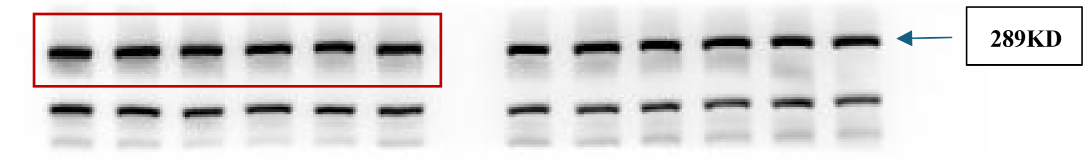

mTOR

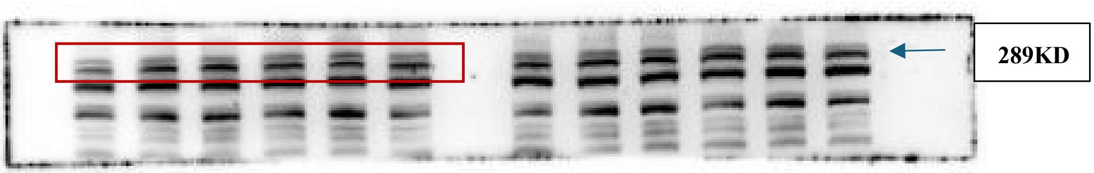

p-PI3K

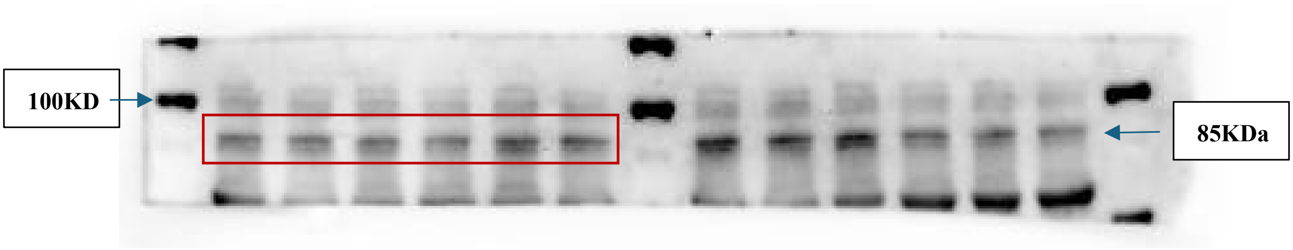

PI3K

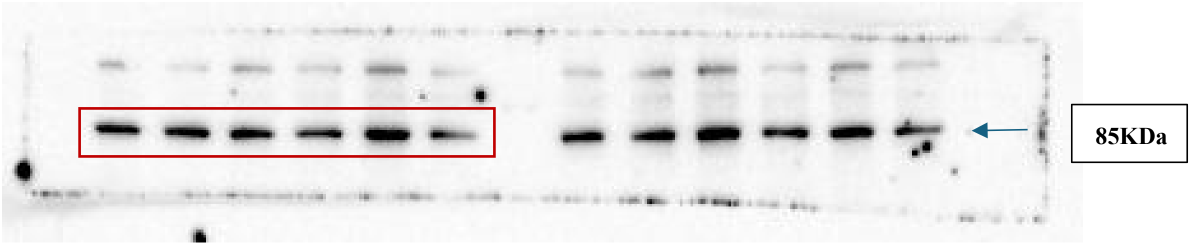

p-Akt

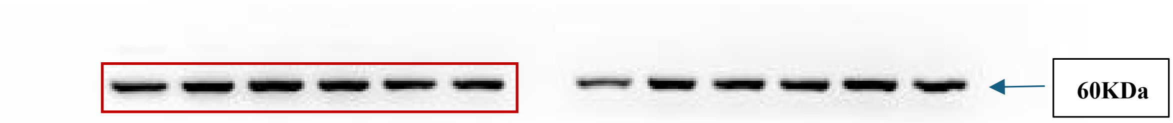

Akt

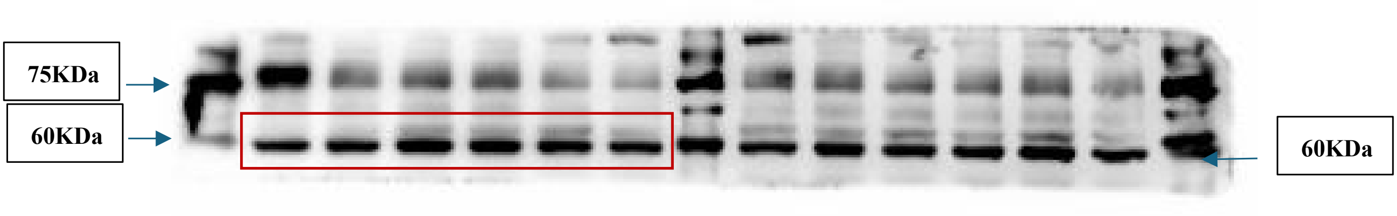

p-JNK

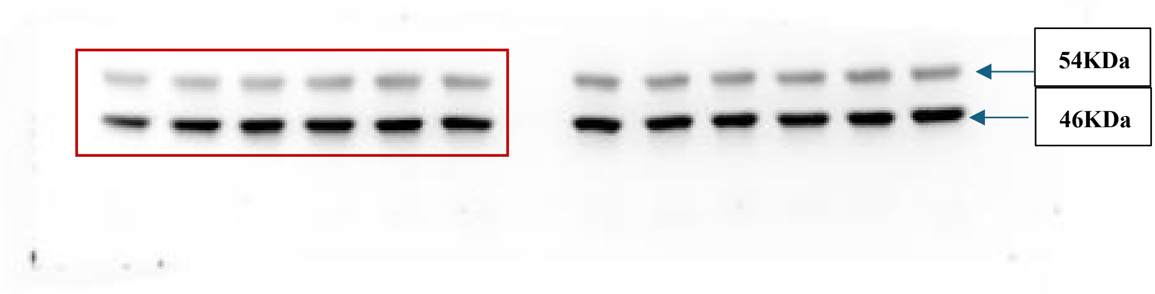

JNK

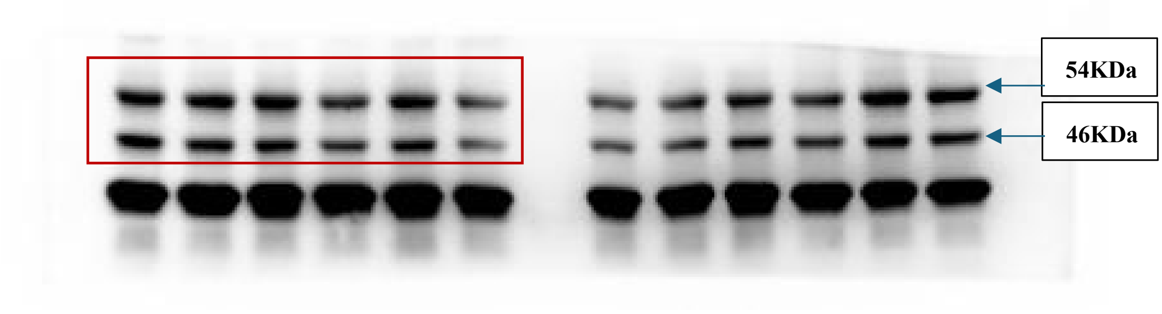

GAPDH

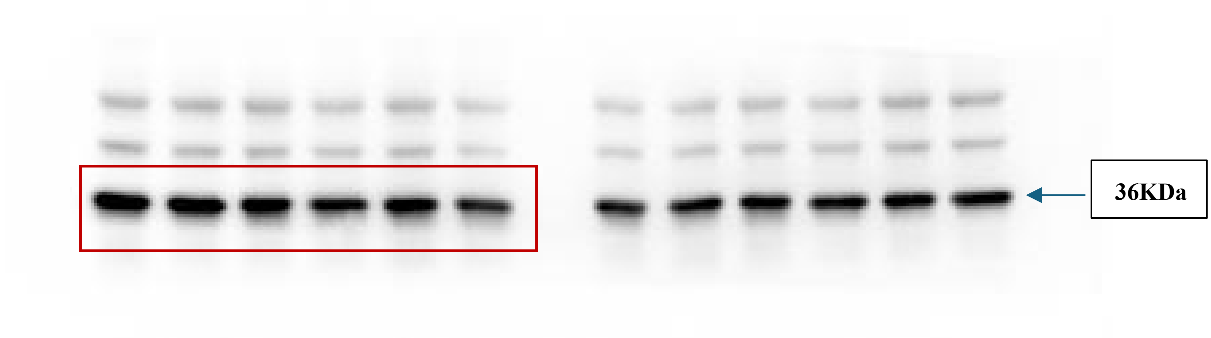

Full unedited blots for Supplementary Figure 6C

p-mTOR

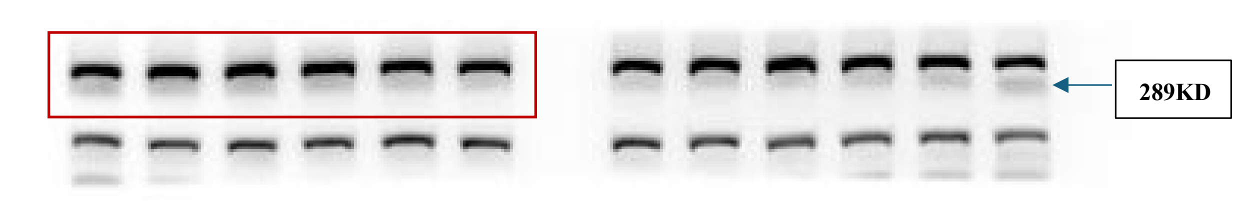

mTOR

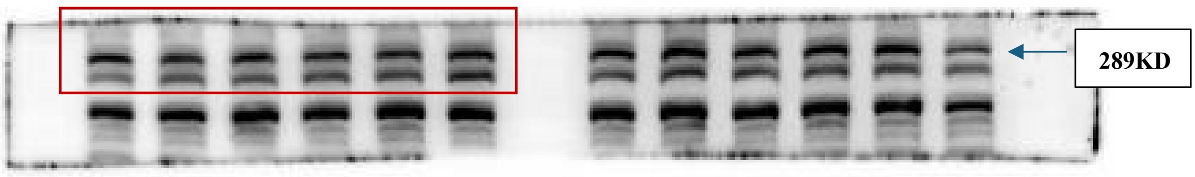

p-PI3K

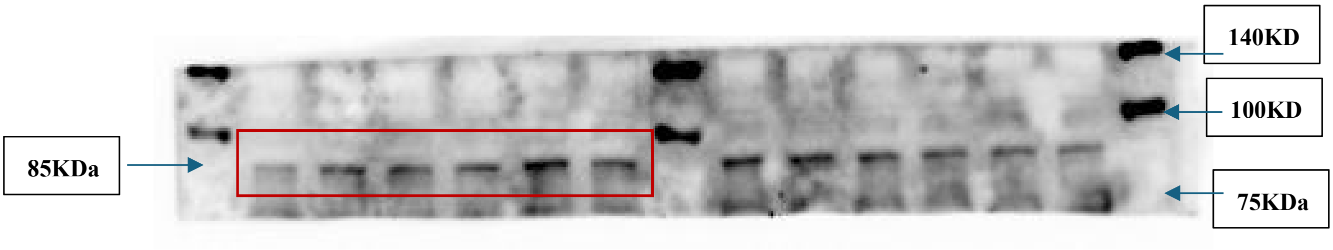

PI3K

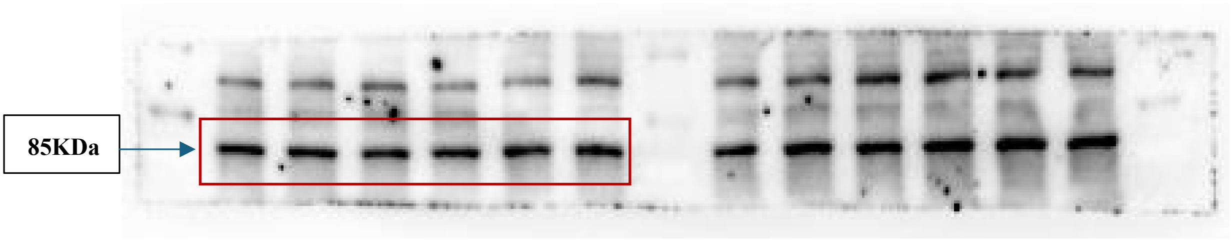

p-Akt

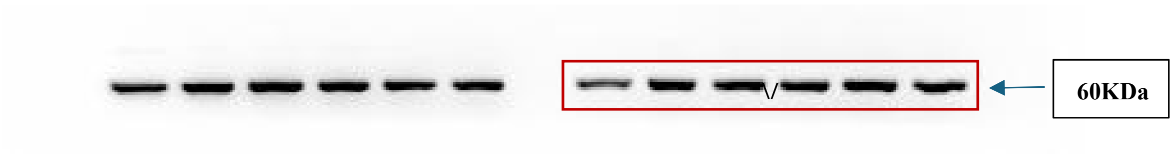

Akt

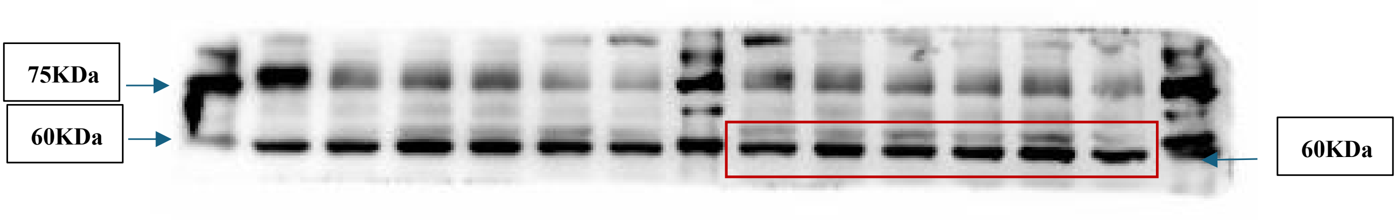

GAPDH

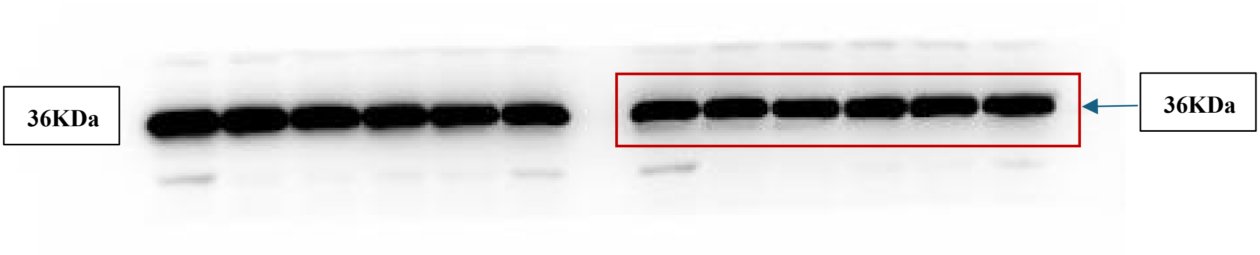

p-JNK

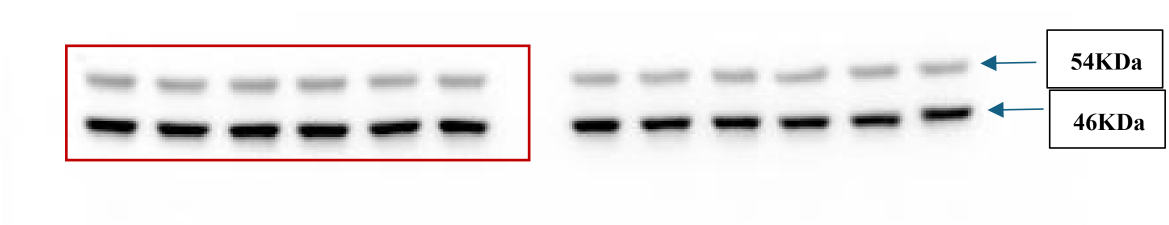

JNK

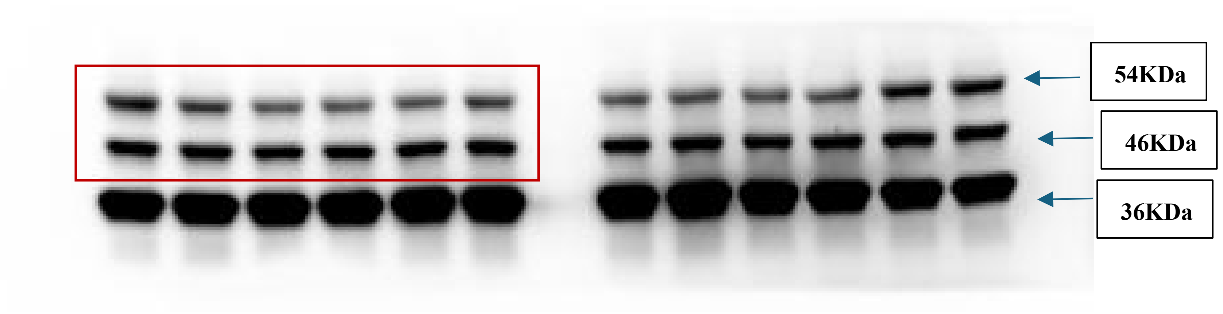

GAPDH

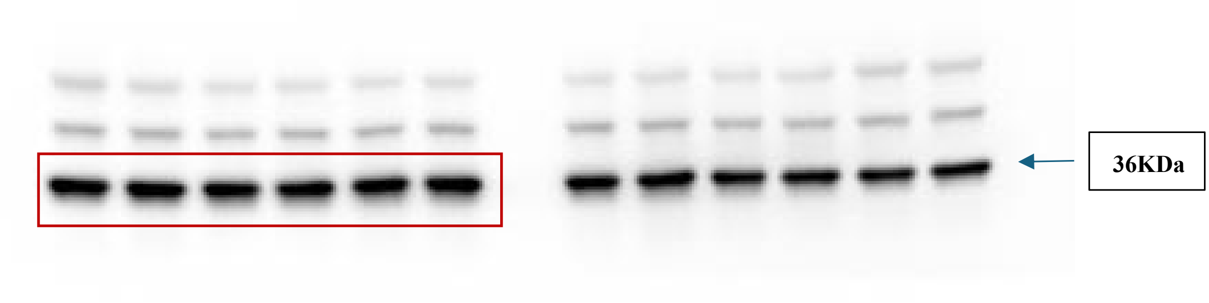

Supplement: Supplementary file 8 — Data S1. Xxxxxx. [file CNS-31-e70368-s003.pdf]
